# An in vitro demonstration of a passive, acoustic metamaterial as a temperature sensor with mK resolution for implantable applications

**DOI:** 10.1038/s41378-023-00632-x

**Published:** 2024-01-17

**Authors:** Lucrezia Maini, Vicente Genovés, Roman Furrer, Nikola Cesarovic, Christofer Hierold, Cosmin Roman

**Affiliations:** 1 Micro- and Nanosystems, Department of Mechanical and Process Engineering, ETH Zurich, Tannenstrasse 3, 8092 Zurich, Switzerland; 2 Translational Cardiovascular Technology, Department of Health Science and Technology, ETH Zurich, Leopold-Ruzicka-Weg 4, 8093 Zurich, Switzerland; 3 Transport at Nanoscale Interfaces, Swiss Federal Laboratories for Materials Science and Technology, EMPA, Überlandstrasse 129, 8600 Dübendorf, Switzerland; 4 Department of Cardiothoracic and Vascular Surgery, Deutsches Herzzentrum der Charite (DHZC), 13353 Berlin, Germany

**Keywords:** Engineering, Physics

## Abstract

Wireless medical sensors typically utilize electromagnetic coupling or ultrasound for energy transfer and sensor interrogation. Energy transfer and management is a complex aspect that often limits the applicability of implantable sensor systems. In this work, we report a new passive temperature sensing scheme based on an acoustic metamaterial made of silicon embedded in a polydimethylsiloxane matrix. Compared to other approaches, this concept is implemented without additional electrical components in situ or the need for a customized receiving unit. A standard ultrasonic transducer is used for this demonstration to directly excite and collect the reflected signal. The metamaterial resonates at a frequency close to a typical medical value (5 MHz) and exhibits a high-quality factor. Combining the design features of the metamaterial with the high-temperature sensitivity of the polydimethylsiloxane matrix, we achieve a temperature resolution of 30 mK. This value is below the current standard resolution required in infrared thermometry for monitoring postoperative complications (0.1 K). We fabricated, simulated, in vitro tested, and compared three acoustic sensor designs in the 29–43 °C (~302–316 K) temperature range. With this concept, we demonstrate how our passive metamaterial sensor can open the way toward new zero-power smart medical implant concepts based on acoustic interrogation.

## Introduction

Implantable medical devices are used in patients mostly to monitor or detect a clinical condition or to perform subcutaneous drug delivery. Depending on how data transmission and energy supply are carried out, implantable devices can be divided into two categories: wired or wireless. Wired solutions have been shown to be more prone to infections and contamination^1–3^ because of the open wound necessary for the cables to connect the implant with a base unit outside the body. In contrast, wireless solutions avoid these complications^4,5^ and have therefore become a goal for medical implantable devices.

Many implantable wireless medical devices rely on electromagnetic (EM) coupling for information transmission and energy supply, with a sensing unit integrated into a resonant circuit. Typically, the resonant frequency proportionally shifts with the variation in the biomechanical or physical parameter of interest that is measured^6–8^. EM coupling comes with some challenges. First, the sensor needs a customized receiving unit, which is not scalable^9^. Second, EM waves are strongly absorbed by the surrounding tissue^10^. Typical working frequencies of EM-based sensors are in the GHz range, which raises concerns for the safety of the patient. Therefore, EM coupling must comply with regulatory standards in terms of power and interrogation time^11^ to limit harmful effects, such as heating, in the human body.

To address some of these issues, “*body dust”* and *“neural dust*”^12,13^ have been recently proposed as highly miniaturized sensors implanted in the body based on ultrasound instead of EM waves. With this approach, it is possible to achieve power levels up to and above 72× higher than that with EM coupling and higher penetration depths in tissues without being harmful^13^. However, these acoustic approaches and more recent ones^14^ are still limited by complex fabrication processes, electronic interfaces for signal transmission, and complex energy management subsystems. Moreover, an active electronic module is usually still needed to allow transmission of the sensor output data. A sensing solution that does not require additional modules to receive and transmit the signal or energy to/from the sensor would be highly desirable, from simpler fabrication and reduced system complexity perspectives.

Metamaterials are interesting candidates for developing passive sensors due to their designable physical properties. They have been designed to control and change the propagation of optical and acoustic waves, to name a few applications. In the realm of optical metamaterials, many intriguing designs have been developed to control thermal properties, even in combination with different physical domains (e.g., *multiphysics metamaterials*)^15–17^. Due to their capabilities to interact with acoustic waves, in particular ultrasound waves, the use of acoustic metamaterials is a promising approach for implantable devices. Manipulation of acoustic waves in an unconventional manner has already been demonstrated^18^, including acoustic “invisibility” or cloaking^19^. Additionally, a recent study has shown the potential of active acoustic metamaterials for drug delivery in the management of acute disease^20^.

In this work, we present a proof-of-concept of an acoustic temperature sensor for medical applications that is purely passive and based on an acoustic metamaterial design. The acoustic properties of the metamaterial sensor are modulated by temperature. Interrogation via an external ultrasound transducer is performed by analyzing the reflected acoustic signal, eliminating the need for energy transfer, storage, and management. The metamaterial consists of two contrasting acoustic impedance materials: silicon micropillars—arranged in a hexagonal lattice—and an embedding polymeric matrix made of polydimethylsiloxane (PDMS). The acoustic metamaterial is fabricated from a 4-inch, 500 μm-thick silicon wafer via deep reactive ion etching (DRIE). Its design is optimized to exhibit an acoustic reflectance whose resonance is close to ~5 MHz, which is a typical central frequency of medical ultrasonic probes^21^.

We investigate in vitro three different designs in the 29–43 °C (~302–316 K) temperature range: a simple bilayer of silicon and PDMS (hereafter Bilayer) and two silicon acoustic metamaterials, without and with a PDMS coating (hereafter Si-Meta and PDMS-Meta, respectively). The ultrasonic metamaterial demonstrated in this study with crucial performance is the PDMS-Meta. The two other designs are used to clarify the physical temperature dependency and relative contribution of PDMS with respect to the silicon pillars. While the Bilayer, whose acoustic response is theoretically understood, is used to explore the temperature behavior of PDMS, the Si-Meta is used to clarify the origin of the acoustic resonances and the role of the silicon pillars in the metamaterial.

We demonstrate, by experiments and simulations, how the temperature sensitivity of the polymeric matrix, complemented by a sharp metamaterial acoustic mode, results in an average resolution of ~30 mK. This resolution has the potential to open new avenues for medical applications by enabling passive monitoring and interrogation of the local intracorporeal temperature at one or multiple locations in the human body.

## Results

### Acoustic design considerations

**Fig. 1:**
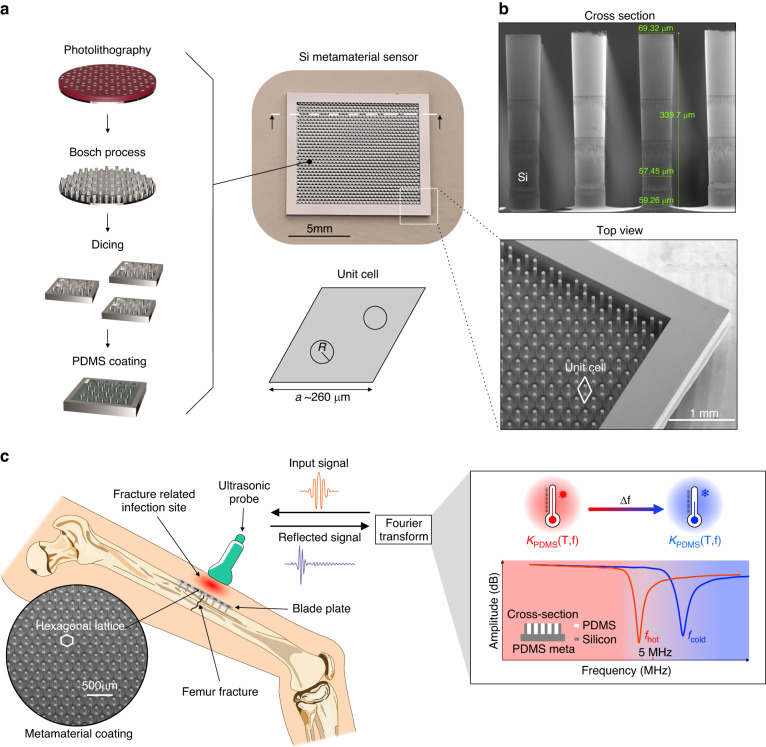
Concept of the acoustic temperature sensor in bone implants. **a** Fabrication process flow starting from a 4-inch, 500 μm-thick silicon (Si) wafer based on deep reactive ion etching (DRIE) and coating of polydimethylsiloxane (PDMS) as a polymeric matrix. **b** Scanning electron microscopy (SEM) details of the fabricated silicon micropillars including measured dimensions (nominal height: 350 μm and nominal radius: 35 μm), and schematic top view of the unit cell. **c** Envisioned application and sensing principle of the acoustic temperature sensor. An ultrasonic probe is utilized to interrogate the sensor located on the implant surface. Temperature variations that change the bulk modulus of the involved materials (PDMS and Si) produce a shift in the resonance frequency of an acoustic mode proportional to the temperature variation

Silicon and PDMS are the two materials involved in the three sensor designs. PDMS was chosen as the embedding and encapsulation material due to its high biocompatibility and demonstrated use in implantable sensors^22–26^, while silicon can be precisely micromachined via DRIE (Fig. 1a; for the detailed fabrication process flow, see the Methods). The acoustic impedance of silicon ( ~ 22 MRayl) is one order of magnitude larger than that of water or soft tissues ( ~ 1.7 MRayl) in the human body^27^. This high acoustic impedance mismatch is crucial to achieve a high signal intensity at the reception since the sensing principle is based on the reflected echo (pulse-echo, P/E) configuration^28,29^.

The two acoustic metamaterials were dimensioned to resonate at 5 MHz, which is a commonly utilized frequency in medical applications^30–32^. The lattice constant (detailed geometrical definition in Fig. 1a, b and Supplementary Fig. 5) of the unit cell was chosen in correspondence with the sound wavelength of 5 MHz in PDMS.

The radius of the two silicon pillars within the unit cell was then adjusted to approach the ultrasonic probe working frequency (Supplementary Fig. 6). At 37 °C (310 K), the Si-Meta exhibits a resonance at 5.05 MHz, while the PDMS-Meta exhibits a resonance at 5.02 MHz (see below). Finally, the PDMS layer in the Bilayer acoustic sensor exhibits a resonance close to 5.12 MHz.

### Experimental design

Relevant clinical values in medical thermography are 36–41 °C ( ~ 279–314 K) for the temperature range and 0.1 K for the temperature resolution^33,34^. We set the temperature range of our experiments to cover a broader range: 29–43 °C ( ~ 302–316 K).

**Fig. 2:**
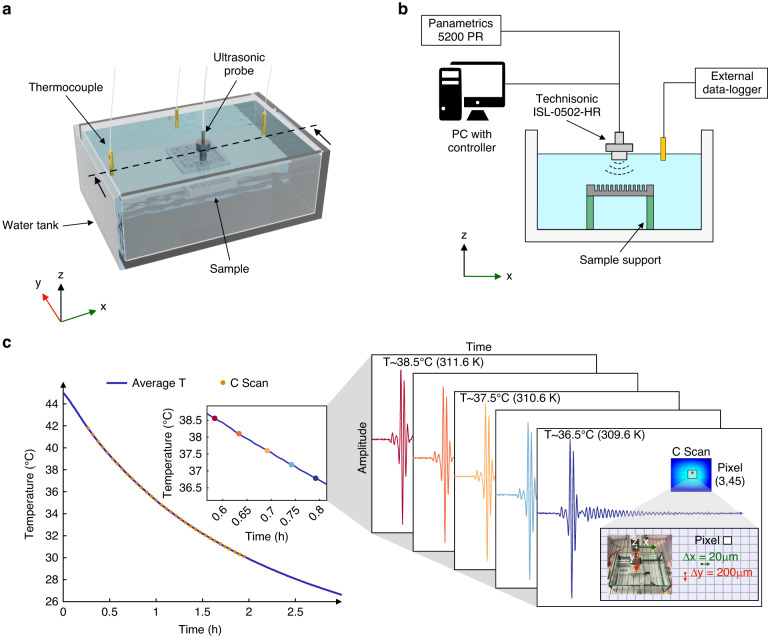
Experimental ultrasonic setup with temperature variation. **a** Three-dimensional view of the ultrasonic temperature setup consisting of three temperature probes connected to an external data logger, a 5-liter water tank, and an ultrasonic probe. The sample is immersed in water, previously heated at 45 °C (318 K) using a kettle. **b** Cross-sectional view (x–z) of the ultrasonic setup along the dashed line in **a**. The sample is supported at the edges. **c** Recorded transient behavior of the temperature in water (average value, purple solid line). The time trace (A-scan) for each pixel is recorded in the ROI (pixel (3,45) displayed in the inset of Fig. 2c). The C-scan consists of the spatial representation of the A-scans in the ROI, displayed as the root-mean-square voltage in the area where it is computed. The process is repeated for every temperature in the experimental range

We heated the water contained in a 5-liter tank^35^ (Fig. 2a) with a kettle up to 45 °C (~318 K). During natural cooling to an ambient temperature of 26.5 °C (~299.7 K), we recorded the temperature in the water tank with a 2–3 min sampling time, depending on the sample (see Methods section).

The different samples, i.e., acoustic sensors, were immersed in the water and supported at the extremities (Fig. 2b). The sensors were interrogated at different locations with a moving stage carrying the probe. A Gaussian pulse with a central frequency of 5 MHz and a duration of ~1 μs (Supplementary Fig. 7) was used as the interrogation signal.

The experimental region of interest (ROI) was defined close to the sample center, with an area corresponding to 1.8 × 1.8 mm^2^. Then, 9 × 90-time traces of the reflected signal (A-scan), with a spatial grid spacing of 20 μm and 200 μm in the *x* and *y* directions (pixel size), were recorded in the ROI (Fig. 2c). Once the A-scans were acquired, the C-scan was built as a spatial matrix of the A-scan signals: the root-mean-square voltage of the time signals obtained in the A-scan is displayed in Fig. 2c (C-scan). This process was iterated for all temperatures in the experimental range of investigation.

### Temperature sensitivity analysis

**Fig. 3:**
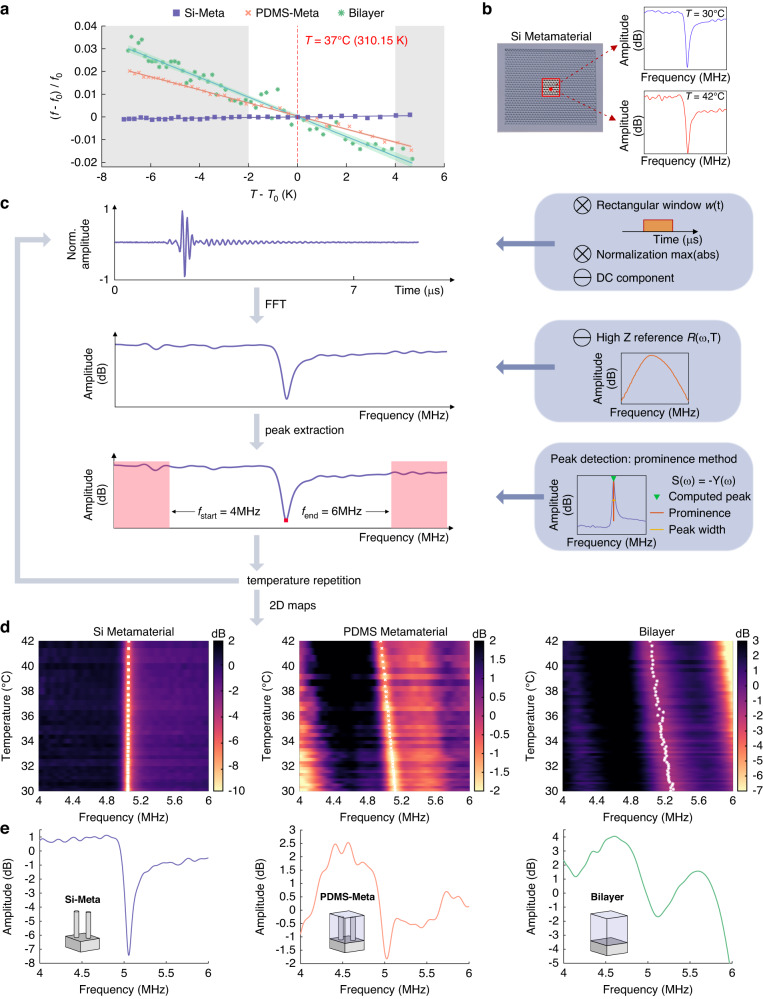
Algorithm for calculating the temperature sensitivity of one pixel in the ROI. **a** Input–output sensor characteristics extracted from experimental resonance frequencies for one example pixel (3,44) for the three acoustic sensors (□ The Si-Meta; **x** The PDMS-Meta; * Bilayer) and the relevant clinical range (white area). **b** ROI (red edges) and FFT amplitudes of the Si-Meta sample for two different temperatures. **c** Steps in the signal processing to extract the frequency peak for a given temperature. In the blue insets, the input operations are represented. **d** 2D representation of the FFT amplitude for pixel (3,44) as a function of temperature. The extracted resonance frequencies at each temperature are overlaid as markers. **e** FFT amplitude at *T* = 37 °C (310 K) for the different sensors

In this section, the temperature sensitivity (*S*) values of the three sensors are compared. The extraction of the sensitivity for each pixel in the defined ROI was performed using the algorithm shown in Fig. 3. The waveform of the reflected signal was recorded for each pixel, its amplitude was normalized, and its average value (DC component) was subtracted before applying a fast Fourier transformation (FFT). To remove the ultrasound transducer spectral response and other influences, the amplitude spectrum (high-impedance reference, high *Z* in Fig. 3c) of an echo signal from a polished, thick aluminum block—an approximation of a perfect reflector—was subtracted on the dB/log scale. An example resulting spectra for the three acoustic sensors at *T* = 37 °C (~310 K) are shown in Fig. 3e. Peak detection with a prominence threshold in a reduced frequency range of 4–6 MHz was subsequently utilized to extract acoustic resonance frequencies. These operations were repeated for each temperature (Fig. 3c).

The amplitude spectra as a function of temperature are summarized as color maps in Fig. 3d, which also contain the extracted acoustic resonance frequencies as overlaid markers. In the (4–6) MHz frequency range, the Si-Meta shows a single, sharp acoustic resonance (see also Fig. 3e). The Bilayer shows two broad resonances, as predicted by the theory of acoustic reflection from multiple layers (see Supplementary Text, Bilayer analytical model and Supplementary Fig. 4). The spectrum of the PDMS-Meta is the most complex, showing both broad peaks and one sharp resonance close to 5 MHz, with the latter being utilized as the sensor signal in the following sections.

Figure 3a shows the input‒output characteristics of the three acoustic sensors extracted from the data in Fig. 3d, where the output is defined as the resonance frequency shift relative to the reference value *ƒ*_0_ at 37 °C (310 K), i.e., $(f-{f}_{0})/{f}_{0}$.

Whereas the Si-Meta resonance shows a low-temperature dependency, the acoustic spectra of the PDMS-Meta and the Bilayer are visibly sensitive to temperature (Fig. 3d, e). The average sensitivities for the three acoustic sensors, extracted by linear interpolation of the input–output characteristics, are 1.4·10^−4^ K^−1^ for the Si-Meta, 2.9·10^−3^ K^−1^ for the PDMS-Meta and 4.4·10^−3^ K^−1^ for the Bilayer. As already observed, the sensitivity of the latter two exceeds that of the pure silicon metamaterial by at least 20 times, suggesting an important role played by the PDMS coating, which will be discussed later. The Bilayer shows a sensitivity 1.5 larger than that of the PDMS-Meta, but the next section will discuss the different performance behaviors with respect to resolution.

### Temperature resolution analysis

The resolution of the three sensors is analyzed in this section. The resolution is determined on the one hand by the sensitivity and on the other hand by the noise, with the latter being dependent on filtering or averaging signal processing operations.

**Fig. 4:**
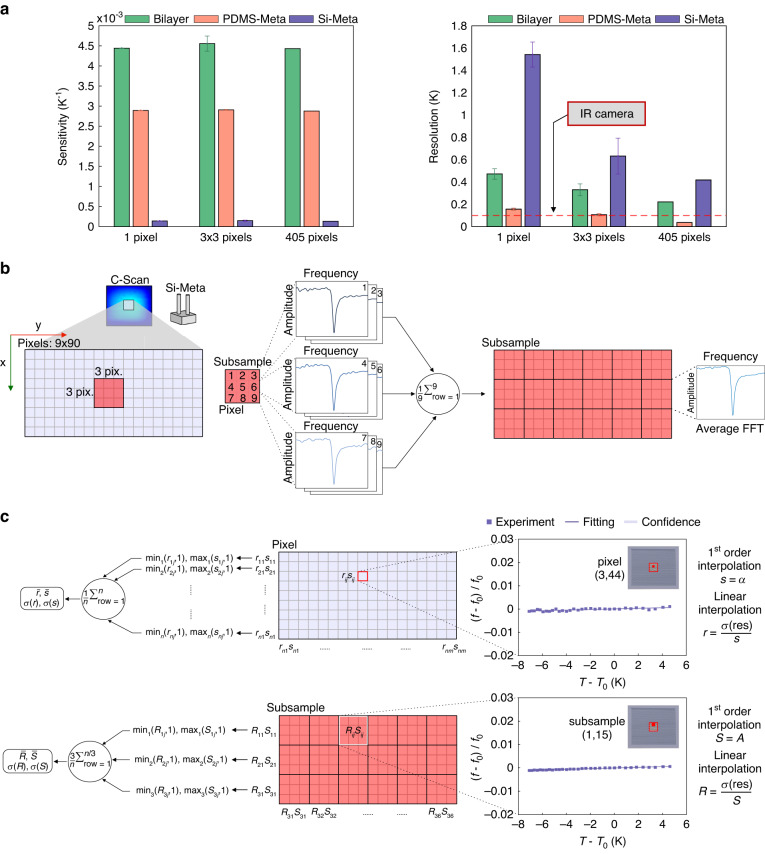
Averaging and extraction of the temperature sensitivity and resolution of the three sensors. **a** Mean temperature sensitivity and resolution per pixel (1 pixel), 3 × 3 pixels, and 405 typical pixels for the different sensors. Dashed line: typical resolution of infrared (IR) cameras used in medical thermometry (see Results: Experimental design). **b** ROI is defined as 9 × 90 pixels (light blue rectangle with 9 × 18 pixels in the figure for simplicity). A subsample (pink square) contains 3 × 3 pixels. The 9 waveforms in each subsample are transformed into spectra as previously described in Fig. 3 and then averaged. The initial ROI (9 × 18 pixel matrix) is thus reduced by averaging to a 3 × 6 subsample matrix. **c** Process for extraction of the sensitivity and resolution, and the standard deviation in one-pixel analysis $(\bar{r},\bar{s})$ and in subsample analysis $(\bar{R},\bar{S})$. For each row of the pixel or subsample matrix defined by the ROI, the minimum (maximum) value of the resolution (sensitivity) is extracted, and the mean and standard deviation are computed (Fig. 4a, first and second bars)

Figure 4 describes the averaging and extraction processes used to determine the resolution and sensitivity of the three sensors. As mentioned before, an ROI of 9 × 90 pixels was defined close to the center of the sample (blue rectangle, Fig. 4b). To assess the influence of spatial averaging, the ROI was divided into subsamples (pink square, Fig. 4b) consisting of 3 × 3 pixels. Then, using the algorithm detailed in Fig. 3, the acoustic spectrum was computed. For each subsample, the 9-pixel spectra were averaged, resulting in a reduced 3 × 30 ROI matrix. Finally, as shown in Fig. 4c, the min. (max.) temperature resolution *r*_*nm*_ (sensitivity *s*_*nm*_) of a single pixel was extracted per row. Afterward, their mean and standard deviation values were computed, resulting in the values displayed in Fig. 4a. Similarly, we extracted the min. (max.) temperature resolution *R*_*ij*_ (sensitivity *S*_*ij*_) of the 3 × 3 pixel subsample per row and calculated their mean and standard deviation. The resolution was extracted as the ratio of the standard deviation of the residuals to the sensitivity (additional details are provided in the Methods section). We implemented this process by discarding 50% of atypical measurement sensitivity values based on the empirical cumulative distribution function, ECDF (see Methods). The third column in Fig. 4a displays the sensitivity and resolution results computed over the typical 50% pixels—equivalently 405 pixels—extracted with the ECDF statistical procedure (Supplementary Fig. 8).

In Fig. 4a, the resulting mean sensitivities and resolutions within the ROI per pixel (1 pixel), per subsample (3 × 3 pixels), and 405 averaged typical pixels are displayed. Sensitivity values are already presented in the previous section, with the Bilayer being 1.5× more sensitive than the PDMS-Meta, which in turn is approximately 20× more sensitive than the Si-Meta. As expected, the subsample averaging process does not significantly affect the temperature sensitivity. However, averaging does improve the resolution by a factor of 5 in the PDMS-Meta (0.16 K vs. 0.03 K, before vs. after averaging), 2 in the Bilayer (0.47 K vs. 0.22 K), and close to 4 in the Si-Meta (1.54 K vs. 0.42 K). With averaging over the typical pixels (405), the resolution is well below the current resolution used in infrared thermometry for medical applications (0.1 K, dashed red line in Fig. 4a).

The PDMS-Meta—upon averaging over the 405 typical pixels—achieves the best temperature resolution, with a value almost one order of magnitude higher in comparison to the Bilayer (0.03 K vs. 0.22 K). The temperature sensitivity is comparable, in order of magnitude, to that of the Bilayer design (2.9·10^−3^ K^−1^ and 4.4·10^−3^ K^−1^), suggesting a role of the PDMS matrix in the temperature sensitivity, as investigated in the next subsection.

As shown in the Supplementary Information (Supplementary Fig. 12 and the Supplementary Text), we computed the signal-to-noise ratio (SNR) and its dependency on temperature. This is an alternative figure of merit for the quality of the signal and the influence of noise in our system.

### Physical explanation of the temperature sensitivity

In this section, possible explanations of the differences in the temperature sensitivity of the three sensors are investigated based on further data analysis in conjunction with finite-element modeling simulation.

The temperature sensitivity of the Si-Meta (1.4·10^−4^ K^−1^) should be related to either the thermal expansion of silicon or its temperature-dependent Young’s modulus (equivalently, the speed of sound). The thermal expansion coefficient of silicon is approximately 2·10^−6^ K^−1,^ ^36^, while its Young’s modulus thermal coefficient is 0.5·10^−4^ K^−1,^ ^37^. Since the latter has the same order of magnitude as the measured sensitivity of the Si-Meta, we propose that the temperature dependency of the Young’s modulus—and thereby of the speed of sound—of silicon is the main contributor to the Si-Meta temperature sensitivity.

The PDMS-based sensors show up to 31× higher sensitivities (4.4·10^−3^ K^−1^ in the Bilayer vs. 1.4·10^−4^ K^−1^ in the Si-Meta) than the pure silicon metamaterial. Furthermore, the PDMS-Meta and the Bilayer have sensitivities on the same order of magnitude, 2.9·10^−3^ K^−1^ and

4.4·10^−3^ K^−1^, respectively. These observations suggest a dependency of the PDMS material properties on temperature as a possible candidate for explaining the thermal sensitivity of the two PDMS-based sensors. The experimental value of the thermal expansion coefficient of PDMS, measured at curing temperatures similar to ours, is 2.8·10^−4^ K^−1,^ ^38^, which is one order of magnitude below our sensitivity values. In a separate experimental study (Supplementary Fig. 2), we characterized the temperature dependency of the p-wave speed of sound in PDMS^39^. From these measurements, we estimate a change of 15% in the PDMS bulk modulus when the temperature is varied in the 27–50 °C (~300–323 K) range (Supplementary Information, PDMS elasticity matrix model, and multiphysics simulation). This corresponds to an approximately 6.5·10^−3^ K^−1^ thermal coefficient, which agrees in magnitude with our sensitivity values. To make this agreement even more precise, we modeled our sensor in COMSOL Multiphysics 6.0.

**Fig. 5:**
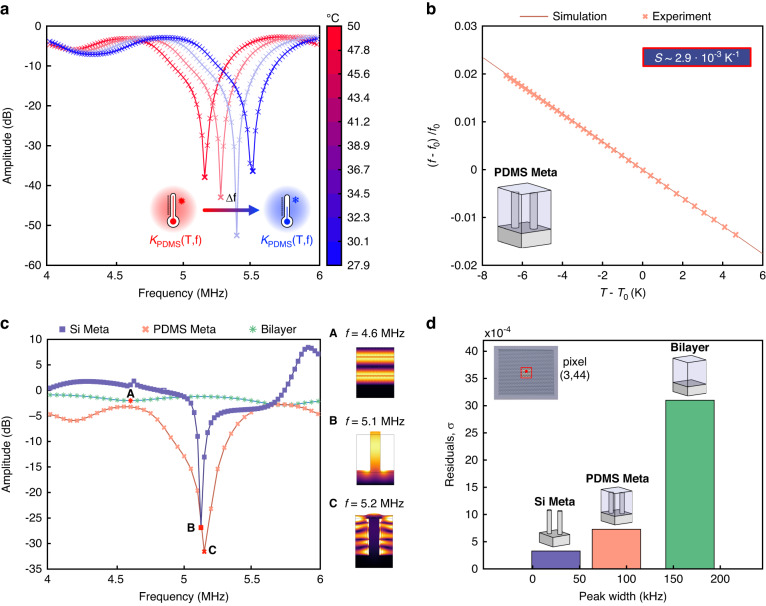
Temperature sensitivity, resolution analysis, and comparison with experiments. **a** Simulated reflection spectra of the PDMS-Meta with variations in the bulk modulus *K*_PDMS_ equivalent to temperature variations. **b** Comparison of simulated (cross markers) and experimental (continuous line) temperature sensitivities. **c** Simulated reflection spectra of the three sensors and acoustic mode shapes at 50 °C (~323 K) (A–C, absolute value of the vertical displacement, where black is equivalent to no displacement, at the resonance frequency). **d** Experimental standard deviation (${\rm{\sigma }}$) of the residuals for pixel (3,44) vs. the average over the temperature of the peak width of the three sensors, computed at −0.5 dB

Simulated spectra of the PDMS-Meta with bulk modulus variations, as induced by temperature changes, are shown in Fig. 5a. A significant shift in the acoustic resonance frequency is visible upon temperature variation, in qualitative agreement with the experimental trend. In Fig. 5b, the extracted relative resonance frequency shifts versus temperature are plotted together with the corresponding experimental values. The PDMS-Meta sensitivity extracted from the simulation is 2.9·10^−3^ K^−1^, in agreement with the experimental sensitivity. This supports the hypothesis that the temperature sensitivity of the PDMS-Meta sensor is caused by the temperature dependence of the bulk modulus in PDMS.

### Relating the temperature sensitivity to the resolution

To investigate the origin of the differences in the resolution of the three acoustic sensors, their acoustic spectra were simulated, as displayed in Fig. 5c. The simulated spectra show qualitative features similar to their experimental counterparts. As described before, the Si-Meta shows a sharp acoustic resonance, the Bilayer shows broad resonances, and the PDMS-Meta shows both one sharp and broad resonances. In Fig. 5c (A–C, and Supplementary Movies 1–3), the displacement fields at the resonance frequency are shown. The Bilayer resonances (A) correspond to successive longitudinal acoustic modes in the PDMS layer. The Si-Meta resonance (B) is essentially the first longitudinal mode of a silicon micropillar, which acts as a resonator. The sharp PDMS-Meta resonance (C) is mainly the result of an acoustic mode within PDMS, as the displacement in the silicon micropillar is almost negligible compared to the surrounding PDMS coating.

It is very important to note at this point that although both (A) and (C) are modes in PDMS, their resonance sharpness, or peak width, significantly differs (Fig. 5c, d). As discussed below, the peak width plays an important role in explaining the different resolutions achieved by the three acoustic sensors. While the broad resonances of the Bilayer are well predicted by the theory of acoustic reflection from multiple layers (Supplementary Text, Bilayer analytical model), the sharp resonance in the PDMS-Meta is not trivial. Based on similar phenomena observed in the literature^40,41^, we speculate that the sharpness of this resonance is due to acoustic mode localization. The acoustic mode shape in Fig. 5c(C) shows that silicon micropillars do not significantly move compared to the surrounding PDMS matrix. The role played by the micropillars is equivalent to a defect, which ultimately spatially confines the acoustic mode (*localization*) and may therefore lead to a sharper resonance.

As illustrated in Fig. 5c, d, the Si-Meta acoustic resonance is the sharpest (PW = 25 kHz at −0.5 dB), followed by the PDMS-Meta (PW = 89 kHz) and finally the Bilayer (PW = 168 kHz). The standard deviation of the residuals, *σ*—which is a measure of noise—and the resonance peak width (PW) are strongly correlated (Fig. 5d). In addition to *σ*, the temperature sensitivity is equally important in determining the temperature resolution.

In Table 1, we repeat the key figures of merit of the three designs. While the average standard deviation of the residuals, $\tilde{\sigma }=R\cdot S$, is strongly affected by the sharpness of the resonance peak, which is a distinguishing feature of the metamaterial-based designs (green cells, Table 1), the sensitivity (*S*) is a feature of the PDMS-based sensors, introduced by the temperature dependency of the bulk modulus (orange cells, Table 1). This shows that the PDMS-Meta achieves the best resolution because it combines both a sharp feature (low $\tilde{\sigma }$) owing to the underneath metamaterial design and a high-temperature sensitivity due to the presence of the PDMS matrix.

**Table 1:**
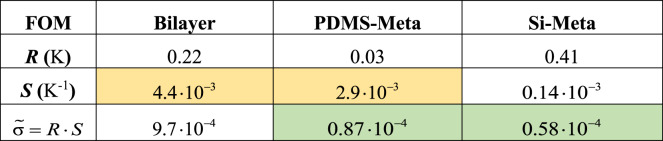
Summary of the relevant figures of merit (FOMs) associated with the three sensors

Orange highlight: PDMS-coated sensors (affected figure of merit: average temperature sensitivity, *S*). Green highlight: Sensors based on metamaterial design (affected figure of merit: average standard deviation of the residuals, $\tilde{\sigma }=R\cdot S$)

## Discussion

In this work, we introduced a new concept for passive temperature monitoring by ultrasonic interrogation based on an acoustic metamaterial sensor. Three different designs for the temperature sensors have been presented, fabricated, and tested. We evaluated their performances with respect to the clinical temperature range and resolution for future potential medical applications.

The temperature sensitivities of the PDMS-Meta and the Bilayer are similar in order of magnitude, both exceeding that of the Si-Meta by a significant factor (>20×). Finite-element simulations support that the origin of the temperature sensitivity is due to the temperature dependence of the bulk modulus of PDMS, which determines the value of the speed of sound of the longitudinal (p-) waves in PDMS. This explains the similar sensitivity values of the Bilayer and the PDMS-Meta sensors.

Concerning the temperature resolution, upon averaging, the PDMS-Meta outperforms the Bilayer by almost one order of magnitude (30 mK vs. 0.22 K) and the Si-Meta by a factor close to 15. We have shown that the peak width is correlated to the standard deviation of the residuals, which ultimately affects the resolution. We explained the higher the PDMS-Meta resolution as arising from a positive synergy of the high-temperature sensitivity of PDMS combined with the underneath silicon micropillar lattice, which leads to a sharp acoustic resonance. We speculate that the sharpness of the resonance is due to an acoustically localized mode caused by the presence of the silicon micropillars. However, the localization mechanism needs to be confirmed through further analysis. Nonetheless, the resonance frequency of the acoustic mode in PDMS is, to a large extent, determined by the geometry of the silicon micropillars (Supplementary Fig. 6). This feature will be useful in designing and tuning acoustic metamaterials for other frequency ranges in the future, depending on the interrogation transducer and the different applications.

Regarding averaging to further improve the resolution, we note that the 405-pixel spatial averaging performed here is probably suboptimal. This averaging procedure is intended to prove that spatial averaging does indeed improve the resolution of all three acoustic sensors. However, the rate of improvement of the resolution with the number of averages is different for each design. The best rate of improvement for the PDMS-Meta was observed to be ~5 for 405 averages. This is still below the highest-expected rate of improvement, which, according to statistics, should be closer to ~20 (√405). Thus, further investigating the limits of spatial averaging and additionally considering temporal averaging has the potential for further temperature resolution improvements. Temporal averaging is particularly interesting because the echo pulses have extremely short durations (~7 μs) compared to the time constant of body temperature variations.

The achieved temperature resolution of ~30 mK of the PDMS-Meta is well below the 0.1 K resolution requirement used in typical medical applications, where infrared (IR) thermography is a common choice. In Table 2, we summarize several implantable temperature sensors in terms of sensing performance and technology. Our sensor compares favorably to other wireless sensors in terms of resolution, and it is only surpassed by optical fiber temperature sensors. However, wired sensors, being transcutaneous, might pose clinical problems related to the development of contamination and infections (see Introduction). Furthermore, we would like to emphasize that the spatial and temporal resolution can still be improved by averaging, as mentioned in one paragraph above.

**Table 2:**
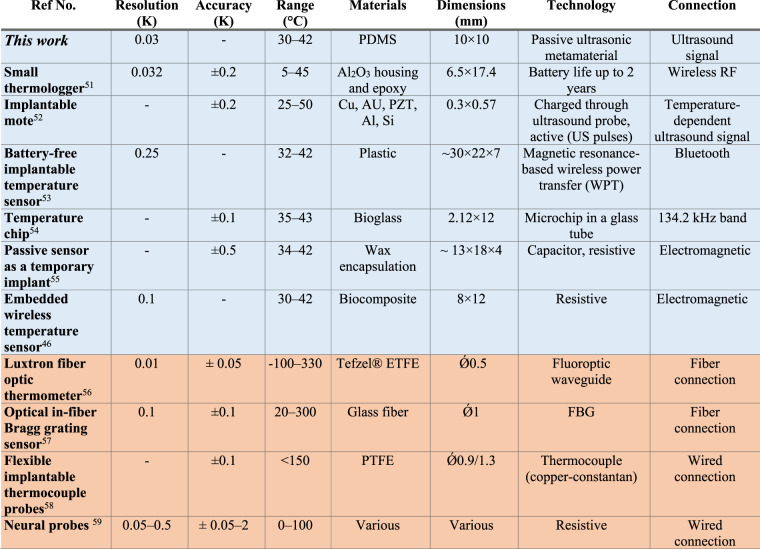
State-of-the-art analysis of implantable temperature sensors^51–55,46,56–59^

Wired sensors are in orange, and wireless sensors are in light blue

Based on the features of our sensor concept, we envision its use in intracorporeal temperature sensing. One example would be monitoring infection processes developing on medical implants. The standard methodologies to diagnose such infections rely on laboratory and microbiological studies, histopathological analysis, and imaging techniques. These approaches have some limitations, including high sensitivity and specificity^42,43^ vs. choice of the sampled area; costs; image artifacts from the prostheses materials; and the amount of radiation introduced into the patient’s body^44,45^. The existing wireless implantable devices^46–48^ are constrained by the limitations listed in the introduction. Infrared (IR) thermography is a new clinical noninvasive strategy for the early detection of infections in bone implants^49,50^. Compared to IR thermography, which is limited to the surface/skin temperature, our implantable, passive acoustic sensors could give access to the local intracorporeal temperature deep inside the human body, with very good resolution.

It is worth mentioning that in this study, we only characterized the sensor in water at a perpendicular incident angle. In the Supplementary Text (fabrication process variations, Supplementary Figs. 10 and 11), we performed Finite Element Method (FEM) acoustic simulations of different case studies focused on fabrication process variations and the acoustic incident angle. In brief, we observed that the resonance frequency exhibits a frequency shift or an amplitude modulation. Nonetheless, these variations are within the capabilities of properly controlled processes. Furthermore, batch sensor calibration could be utilized to remove residual device-to-device variations, as a common practice employed in industry for commercial microfabricated transducers.

Because the sensor signal is based on a resonance frequency shift mechanism, we anticipate that our sensor concept will suffer less from typical scattering mechanisms, which lead mostly to signal amplitude degradation. In the Supplementary Text (see also Supplementary Fig. 13), we have explored by simulation one potential impact caused by the presence of soft tissue in in vivo environments, namely, attenuation. Indeed, we find that whereas the time-domain SNR degrades by a factor of 2 (−6 dB), the resonance frequency-based resolution degrades by a factor of only 1.4.

Future work will be focused on design and material optimization, integration of the metamaterial onto titanium prostheses, cross-sensitivity studies (e.g., strain), and in vivo characterization and demonstration. We believe that this new sensing concept based on ultrasonic metamaterials opens new ways for passively sensing temperature, with great potential in wearable implants, particularly for early detection of infection in implantable prostheses.

## Materials and methods

### Sensor fabrication

The samples were fabricated at the Binning and Roher Nanotechnology Center (IBM-Rüschlikon) from a < 100 > 4-inch p-doped, 500 μm-thick silicon wafer. The wafer was initially baked on a hotplate at 120 °C for 10 min and then treated with HDMS to promote photoresist adhesion. The substrate was coated with 7 μm-thick AZ4562 positive photoresist (Microchemicals GmbH) and soft baked on a hotplate at 120 °C for 3 min. The photoresist was then exposed in hard contact mode to ultraviolet (UV) light of 11 mW/cm^2^ intensity using a photolithographic mask and developed in AZ400K (1:3) solution.

To fabricate the high-aspect-ratio pillars, the unexposed silicon substrate was etched by deep reactive ion etching (DRIE) with alternating SF_6_/C_4_F_8_ for a total of 400 cycles. The residual photoresist was removed by dipping the wafer in acetone and isopropanol, followed by plasma ashing cleaning (700 W, 10 min). Before dicing the wafer into single devices, the substrate was coated by a protective AZ4562 photoresist (7 μm thickness, soft baked at 110 °C for 3 min). Once removed, the Bilayer and the PDMS-Meta devices were coated with 10:1 liquid PDMS (Sylgard 184, Sigma‒Aldrich Schweiz). This coating was realized by dispensing 1 ml of PDMS with a pipette on top of the sensors and using a spin-coater to uniformly distribute the polymer (WS-650MZ-23NPP, 1000 rpm 60 s). The samples were cured in an oven at 70 °C for 1.5 h. The Bilayer was fabricated with the same steps as the PDMS-Meta, but a different photolithographic mask was used to perform the etching.

### Experimental setup

The experimental setup consisted of a broadband, point focus, immersion transducer (Technisonic, ISL-0502-HR, SN: C2315) utilized to generate pulses and record the echoes. The shape of the interrogation signal was a Gaussian pulse with a central frequency of 5 MHz (Supplementary Fig. 7). The transducer was triggered by an ultrasonic pulser receiver (Olympus, 5072PR, P/E settings: Supplementary Table 1) attached to a three-axis mechanical scanner, actuated by a step motor (National Instruments, MID-7604) and controlled by customized LabView code to record 3D ultrasonic datasets. The probe was mounted on an x-y-z scanner. The different samples, i.e., acoustic sensors, were immersed in water and supported at the extremities (see Fig. 2).

We used three thermocouples connected to a data logger (Onset HOBO ux120–006) to record the temperature in the water tank. The temperature was acquired every 30 s, and we averaged the temperature of the three thermocouples. Each C-scan ultrasonic image had a temporal duration of 2–3 min, depending on the sample. Based on the time stamps of the ultrasonic images, we selected the corresponding temperature recordings within the same time window and averaged them. These are the temperature values reported in Fig. 2c, which define the temperature for a given C-scan experiment. This temperature extraction procedure will introduce a small unknown temperature offset in each pixel. However, this offset is systematic because the pixels are scanned in the same order. Therefore, this will only affect temperature trueness and not the temperature resolution, which is our main quantity focused on in this study.

### Sensitivity and resolution calculation

The signal processing and data analysis in this work were implemented in MATLAB R2019a. The calculation of the temperature resolution was implemented in two steps. First, the temperature sensitivity (*S*) was computed as the linear coefficient of the 1st-order interpolation of the relative change $(\tilde{f})$ in the acoustic resonance frequency:

1 $$\tilde{f}=\frac{\varDelta f}{{f}_{0}}=\frac{f(T)-{f}_{0}}{{f}_{0}}$$

where ƒ_0_ is the frequency peak extracted at $T={T}_{0}=37^\circ {\rm{C}}$ (310 K) and $f(T)$ is the resonance frequency measured at other temperatures.

The differential temperature $\tilde{T}$ is defined as:

2 $$\tilde{T}=T-{T}_{0}$$

where *T* is the average temperature corresponding to the specific spectrum.

As an evaluation of the quality of the fitting, the residuals were computed, $\hat{r}=\,{\tilde{f}}_{exp}-{\tilde{f}}_{fit}$, as were their standard deviation *σ*. Temperature resolution *R* was extracted from the standard deviation *σ* of the residuals divided by the temperature sensitivity *S*.

The above analysis was performed on 50% of pixels that had typical sensitivity values, which means that the remaining 50% of the pixels with atypical sensitivity values were excluded from the temperature sensitivity and resolution calculation. Practically, the empirical cumulative distribution function (ECDF) with respect to the sensitivity values was computed (subsamples and pixels per row), and the typical pixels were defined as those with values in the 25–75% range (Supplementary Fig. 8). The resolution and sensitivity values plotted in Fig. 4a (third column) were computed over the average values of these typical pixels. Atypical pixels appear closer to the edge of the selected area within the sample. We suspect these to be mostly due to fabrication defects, such as missing or broken silicon micropillars.

### Mechanical-acoustic simulation

The mechanical-acoustic simulation was implemented in COMSOL Multiphysics 6.0. To compute the reflection spectra (Fig. 5a-c), we simulated the 2D unit cell (fixed bottom boundary) in a water domain. The solid domain of the unit cell (silicon and PDMS) was defined in solid mechanics physics, while water was defined in pressure acoustics physics. The solid mechanics and pressure acoustics physics were coupled with an acoustic-structure boundary condition at the interface between the unit cell and the surrounding medium. We set periodicity conditions at the edges of the simulated domain (Floquet periodicity), and the direction of the k-vector was set to (0,0) for perpendicular excitation. At the top and bottom of the water domain, we defined perfectly matched layers (rational scaling factor and curvature parameter equal to 1). The study was implemented in the frequency domain (frequency step resolution of 20 kHz).

The properties of PDMS were expressed through the definition of a customized isotropic material. First, we introduced a new linear elastic material subnode to distinguish silicon material properties (polysilicon, in the built-in COMSOL library) from those of PDMS. Then, in the PDMS elastic material definition, we set the solid model as isotropic and specified it with respect to the bulk and shear moduli, which are frequency-dependent. To simulate the temperature dependency, we swept the relative change in the elastic moduli in the 0–15% range, with an increment of 5% (Supplementary Text: Multiphysics simulation).

The incident pressure field was defined in the upper water domain as plane waves with amplitude *P*_0 _= 1 μPa:

3 $${P}_{{\rm{in}}}={P}_{0}\cdot {e}^{-i{k}_{0}\cdot cos\theta \cdot y}$$

where $\theta ={0}^{^\circ }$ is the incident angle and ${k}_{0}$ is the *k*-vector computed with the internal COMSOL function.

The frequency of the incoming plane wave was varied in a parametric sweep in the frequency domain study (Supplementary Text: Multiphysics simulation).

The maximum size of the mesh was defined to satisfy the acoustic wavelength in the material divided by a factor of 6.

The amplitude of the reflection spectrum was computed at the top boundary interface of the sensors as:

4 $${|{\rm{R}}|}_{{\rm{dB}}}=20\cdot {\log }_{10}\left|\frac{{P}_{s}}{{P}_{{\rm{in}}}}\right|$$

where ${P}_{s}$ is the scattered pressure.

To validate the simulation results in the PDMS-Meta, we first compared the analytical solution of the Bilayer structure with the corresponding simulated model in which the customized material properties of PDMS were defined (Supplementary Fig. 4 and Supplementary Table 2).

### Supplementary information

Supplemental Material

Supplementary Movie 1

Supplementary Movie 2

Supplementary Movie 3

## Data Availability

The data that support the findings of this study are available in the ETH Zurich research collection.
