# Supplementary material for: An in vitro demonstration of a passive, acoustic metamaterial as a temperature sensor with mK resolution for implantable applications
Source: Microsyst Nanoeng. 2024 Jan 17;10:8. doi: 10.1038/s41378-023-00632-x (PMC10794229; doi:10.1038/s41378-023-00632-x)
Supplement: Supplementary file 1 — Supplemental Material [file 41378_2023_632_MOESM1_ESM.docx]

Supplementary Information for

An in-vitro demonstration of a passive, acoustic metamaterial as a temperature sensor with mK resolution for implantable applications

*Lucrezia Maini,^1^* Vicente Genovés,^2^ Roman Furrer,^3^ Nikola Cesarovic,^2,4^
Christofer Hierold,^1^ Cosmin Roman^1^*

^1^Department of Mechanical and Process Engineering, ETH Zurich, Zurich, 8092, Switzerland.

^2^Department of Health Science and Technology, ETH Zurich, Zurich, 8093, Switzerland.

^3^Swiss Federal Laboratories for Materials Science and Technology, EMPA, Dübendorf, 8600, Switzerland.

^4^Department of Cardiothoracic and Vascular Surgery, German Heart Center Berlin, Berlin, 13353, Germany.

*Corresponding author. Email: [lucrezia.maini@micro.mavt.ethz.ch](mailto:lucrezia.maini@micro.mavt.ethz.ch)

**This PDF file includes:**

Supplementary Text

Figs. S1 to S13

Tables S1 to S4

Movies S1 to S3

References

Supplementary Text

**PDMS elasticity matrix model**

Polydimethylsiloxane (PDMS) can be described as an isotropic linear elastic material ^1^.

The isotropic elasticity tensor for PDMS can be expressed as follows^1^:

$$\left(\begin{matrix} \sigma_{1} \\ \sigma_{2} \\ \sigma_{3} \\ \sigma_{4} \\ \sigma_{5} \\ \sigma_{6} \end{matrix}\right)=\left(\begin{matrix} C_{11} & C_{12} & C_{12} & 0 & 0 & 0 \\ C_{12} & C_{11} & C_{12} & 0 & 0 & 0 \\ C_{12} & C_{12} & C_{11} & 0 & 0 & 0 \\ 0 & 0 & 0 & C_{44} & 0 & 0 \\ 0 & 0 & 0 & 0 & C_{44} & 0 \\ 0 & 0 & 0 & 0 & 0 & C_{44} \end{matrix}\right)\left(\begin{matrix} \varepsilon_{1} \\ \varepsilon_{2} \\ \varepsilon_{3} \\ \varepsilon_{4} \\ \varepsilon_{5} \\ \varepsilon_{6} \end{matrix}\right)$$

where $C_{ij}$are the coefficients of the elasticity tensor. The knowledge of the two independent elastic constants $C_{11}$, $C_{44}$ allows to univocally express the elasticity matrix of the material since $C_{12}=C_{11}-2C_{44}$ .

The independent elasticity constants can be expressed as frequency-dependent quantities in function of the complex speed of sound^2^ ($\hat{c}_{j}$):

$$\hat{C}_{ii}\left(f\right)=\text{ }\rho_{\text{P}}\hat{c}_{j}^{\text{ }2}\left(f\right)$$

where $\rho_{P}$ is the density of the material and $\hat{c}_{j}$ is the complex speed of sound (for longitudinal waves: $i=1, j=L$ and shear waves:$i=4$ , $j=S)$. The complex speed of sound is a frequency-dependent quantity with a real part, the modulus $c_{i}$ , and an imaginary part $\alpha_{i}$ related to the frequency-dependent attenuation^2^:

$\hat{c}_{i}^{\text{ }}\left(f\right)=\frac{2\pi f}{2\pi f/c_{i}+j\alpha_{i}}$ with $\text{ }i=\text{L or S}$

The notation *i* in Supplementary Equation (3) refers to a longitudinal wave (L) or a shear wave (S).

The independent elastic constants used in this work have been linearly interpolated from experimental values^2^, as shown in Supplementary Fig. 1. We chose for the complex elastic coefficients as well as for the PDMS density the experimental values matching the cross-link ratio of the PDMS in our samples^2^ (10:1, $1030\mathrm{kg}m^{-3}$) .

From the literature values at room temperature of the complex-frequency dependent elastic constants^2^, it is possible to express the bulk modulus and shear modulus of PDMS as frequency-dependent complex quantities:

$$\hat{B}\left(f\right)=\hat{C}_{11}\left(f\right)-\frac{4}{3}\hat{C}_{44}\left(f\right)$$

$$\hat{G}\left(f\right)=\hat{C}_{44}\left(f\right)$$

**Multiphysics simulation**

We simulated temperature variations by varying independently the bulk modulus and shear modulus of PDMS.

First, we estimated the interval of percentage variation from experimental values of the longitudinal velocity in PDMS (Supplementary Fig. 2) in the range ($50\div27$)$℃$, while the system is cooling.

Because of the vertical direction of excitation of the incident acoustic beam, we expect that the acoustic resonant modes are propagating mostly longitudinally.

Since the speed of a longitudinal wave in a bulk solid can be expressed as:

$$c_{L}=\sqrt{\frac{B+\frac{4}{3}G}{\rho}}$$

and in PDMS the shear modulus is three orders of magnitude smaller than the bulk modulus (GPa vs MPa)^2^, the contribution of the shear modulus in Supplementary Equation (6) can be neglected.

Because the experimental values of longitudinal speed of sound in PDMS is $970 m s^{-1}$ at $50 ℃$ and $1040 m s^{-1}$ at 27$℃$ (Supplementary Fig. 2), the overall percentage bulk modulus variation in the experimental temperature range is equal to:

$$\Delta B \%=\frac{B\left(27\text{°C}\right)-B\left(50\text{°C}\right)}{B\left(50\text{°C}\right)}=\frac{c_{\text{L}}\left(27\text{°C}\right)^{2}-c_{\text{L}}\left(50\text{°C}\right)^{2}}{c_{\text{L}}\left(50\text{°C}\right)^{2}}=15\%$$

The complex frequency-dependent properties of PDMS have been included in the simulation in the solid mechanics physics, specifically in the linear elastic material section, by expressing the bulk modulus and shear modulus.

The percentage variation of the bulk modulus and shear modulus has been implemented as follows:

$$\hat{B}\left(f\right)=\hat{C}_{11}\left(f\right)-\frac{4}{3}\hat{C}_{44}\left(f\right)+\alpha\cdot\left(\hat{C}_{11}\left(f\right)-\frac{4}{3}\hat{C}_{44}\left(f\right)\right)$$

$$\hat{G}\left(f\right)=\hat{C}_{44}\left(f\right)+\beta\cdot\hat{C}_{44}\left(f\right)$$

where $\alpha$ and $\beta$ are adimensional values (%), varied in the range between 0 and 15; $f$ is the frequency parameter swept in the frequency simulation. The impact of the variation of the bulk modulus and shear modulus has been investigated independently.

As expected from literature values, the influence of the shear modulus is negligible for what concerns the frequency position of the resonant peak of the metamaterial, affecting mostly the value of the amplitude of the reflection spectrum (Supplementary Fig. 3).

The reflection spectrum was extracted at the upper boundary of the unit cell and computed as the average of the ratio between the scattered pressure field and the background pressure field. Then we applied the modulus function on the computed reflection spectrum and transformed it in decibel scale.

The materials used in the simulation are: *water, liquid* (imported from the Comsol Multiphysics built-in library); a customized definition of silicon (Supplementary Table 2a) and PDMS with the aforementioned complex parameters (Supplementary Table 2b).

The mesh has been implemented considering the longitudinal speed of sound in each material domain. In particular, for the water domain, a free triangular mesh has been chosen with maximum element size equal to ${c_{0}}/f/N$, where $c_{0}$ is modulus of the speed of sound of longitudinal waves in water ($1480 m s^{-1}$) and *N* is a conservative factor, which we assumed equal to 6.

For the bilayer and the silicon reference and metamaterial, we used the same procedure and we set the maximum element size of the solid mechanics domains equal to ${c_{2}}/f/N$, with where $c_{2}$ is the modulus of the speed of sound of longitudinal waves in PDMS ($1087 m s^{-1}$). A careful consideration has to be done in the PDMS-Meta sensor since shear waves, even if with a very small amplitude, can be generated at the interface between the silicon pillars and the PDMS matrix. In order to be able to take into consideration this phenomenon, we set a finer mesh along these boundaries through the definition of an edge mesh. The maximum element size on this mesh is equal to ${c_{s}}/f/N$, where $c_{s}$ is the modulus of the speed of sound of shear waves in PDMS^2^ ($100 m s^{-1}$). Finally, we defined a boundary layer mesh in the PDMS domains with a number of layers equal to 20.

For the perfect matched layer, we used a mapped mesh with number of elements equal to 8.

A detailed description of the geometry of the unit cells can be found in Supplementary Fig. 5 and Supplementary Table 3.

**Bilayer analytical model**

In order to validate the simulation results of the PDMS-Meta, we compared the results of the simulated unit cell of the bilayer structure with the well-known literature analytical solution^3^.

The analytical solution was computed for a multilayered structure of finite thickness: a PDMS layer ($350 \mu$m) on top of a silicon layer ($150 \mu$m), in water. Because of the perpendicular interrogation in the experiments, we assumed in the analytical model only longitudinal waves propagating in the system.

Attenuation and frequency dependency of the PDMS properties were taken into account with the complex definition of the longitudinal speed of sound (Supplementary Equation 3).

The analytical results were compared with the FEM computed reflection spectrum of a unit cell simulated in the mechanic-acoustic domain with the same geometrical dimensions (Supplementary Fig. 4).

The relation between the frequency spacing and the thickness of the top PDMS layer can be expressed with the formula for homogeneous layers^3^:

$$f=\frac{\left(n+1\right)}{2}\cdot\frac{c}{d}$$

where c is the longitudinal speed of sound in the material and d the thickness.

**Fabrication process variations**

Because potential discrepancies between the fabricated sensors and the design can be introduced as result of fabrication process variations, their effect on the reflection spectrum was investigated by simulation. In this study, to emphasize the effects of the fabrication process, we have considered intentionally variations which are much larger in comparison to the experimental fabrication process.

Three cases were investigated (see Figs. S10-S11):

- Variation of the incident angle $\theta_{in}$ ($0^{^{\circ}}:5^{^{\circ}}:{10}^{^{\circ}}$);
- Variation of the thickness of the top PDMS layer $t_{\mathrm{PDMS}}$ ($20 \mu m :10 \mu m :40 \mu m$);
- Variation of the height of the Si pillar $l_{\mathrm{pillar}}\equiv h_{\mathrm{PDMS}}$ ($420 \mu m :30 \mu m :480 \mu m$).

A variation of $t_{\mathrm{PDMS}}$ of ±$10 \mu m$ from the value shown in the main manuscript ($30 \mu m$) affects mostly the amplitude of the reflection spectrum at the resonance frequency, with an amplitude modulation of $\sim$10 dB. While the temperature sensitivity is expected not to be affected because the peak locations stays constant, for the temperature resolution further investigation is needed as the sharpness of the peak is affected.

The position of the resonance peak is affected mostly by incident angle perturbations and variations in the height of the silicon. In the former, the amplitude of the resonance peak is reduced by almost $\sim$25 dB with a change $\Delta\theta_{in}= 5^{^{\circ}}$ (see B, Fig. S11-a); new artifact peaks ascribable to interferences effects with the tilt of the incident angle appear in the spectrum (see A, Fig. S11-a).

A height variation of 6% its original value, generates a relative shift in the resonance frequency with a similar 6% magnitude (Fig. S11-c).

**Signal-to-Noise ratio analysis**

In the main text, we have utilized extensively resolution values to compare the different sensor designs. Another measure of the quality of sensor signals is the Signal-to-Noise (SNR) ratio. We have analyzed the SNR and its dependence of temperature for the PDMS-Meta Sample 1. The results of this analysis are shown in Fig. S12. The SNR is defined as:

$$\text{SNR}=20\cdot\log_{10}\left(\frac{S}{\sigma}\right)$$

where *S* is the signal amplitude, defined as the maximum absolute value of the echo signal (See Fig. S12.a), and *σ* is the standard deviation of the noise. To calculate *σ*, a signal-free region preceding the echo pulse was selected (see inset of Fig. S12.a). The SNR was computed for all temperatures and is shown in Fig. S12.b. Its median value is 52dB, and there is a weak dependence of temperature, with a slope of -0.26 dB/K. This decrease correlates with the decrease in the signal amplitude *S* with increasing temperature, with a slope of 20 mV/K, which is visible in Fig. S12.d. We speculate that this decrease in the signal amplitude is related to the increase in the attenuation of water with increasing temperature. On the other hand, the noise variance seems to be temperature-independent (see Fig. S12.c), with a median value of 1.8 mV. Some larger noise variance is visible at some temperatures (e.g. just below 36°C), which warrant additional investigations. The noise temperature-independence suggests that the noise is coming from the read-out electronics mainly.

**Simulated tissue attenuation**

As the final intent of our sensor is to be implanted, an important consideration is the signal degradation due to the presence of soft-tissue (muscle or fat) interposed between the ultrasound probe and the implanted passive sensor. Typical values for soft tissue attenuation coefficients are about -0.6dB/MHz/cm^4^. Considering our operation frequency of 5 MHz and a 1cm-thick soft-tissue, this leads to an expected total attenuation A= -6dB (the US pulse has to traverse the tissue in both directions to reach back the US probe). This attenuation will reduce the SNR computed in the previous paragraph by 6dB.

Our sensor concept involves reading out the temperature from the resonance frequency of a metamaterial in acoustic mode. This is an indirect readout which involves FFT-ing the signal and detecting the resonance peak. Because of these signal processing steps, we have to simulate the effect that a -6dB attenuation in the time-domain would have on the resonance frequency resolution. To do that, we have introduced additive Gaussian noise to the measured signal of the (3,44) pixel at 37°C, such that the initial SNR is reduced by 6dB:

$$y(t)\to y(t)\text{+2}\cdot\sigma\cdot Z$$

where Z is the i.i.d. noise sampled from a standard normal distribution, and *σ* is the measured standard deviation of the noise as described in the previous section. The factor 2 in front of *σ* will produce the desired reduction in the SNR by 6dB. Fig. S13 is showing the initial spectrum (black) along with four random samples of the perturbed spectra (blue). The close-up in Fig. S13.b shows that introduced Gaussian noise in the time domain leads to a randomization of the extracted resonance frequency. In total we performed 10´000 randomization trials (noise injections). This allowed us to recover the average resonance frequency to within 0.65Hz (<0.1ppm error). The computed standard deviation of the resonance frequency *σ_f_* was found to be 2.44kHz.

Based on the simulated resonance frequency fluctuation we can predict that the pixel resolution will degrade from 0.16K (see Fig. S9.f) to 0.23K when the tissue attenuation is -6dB:

$$R\to\sqrt{R^{2}+\left(\frac{\sigma_{f}}{S}\right)^{2}}$$

Extrapolating to the 405 pixel average the resolution is expected to degrade by the same factor, leading to a predicted resolution of 43mK (0.23/0.16∙30mK) from 30mK. Inter alia, this prediction aligns well with the common premise that reading out resonances in the frequency-domain is more robust to noise perturbations than time-domain direct reading. Whereas the time-domain SNR degrades by a factor of 2 (-6dB), the resonance frequency-based resolution degrades by a factor of 0.7 only. For a summary of comparison of the sensor resolution with and without attenuation see Supplementary Table 4.


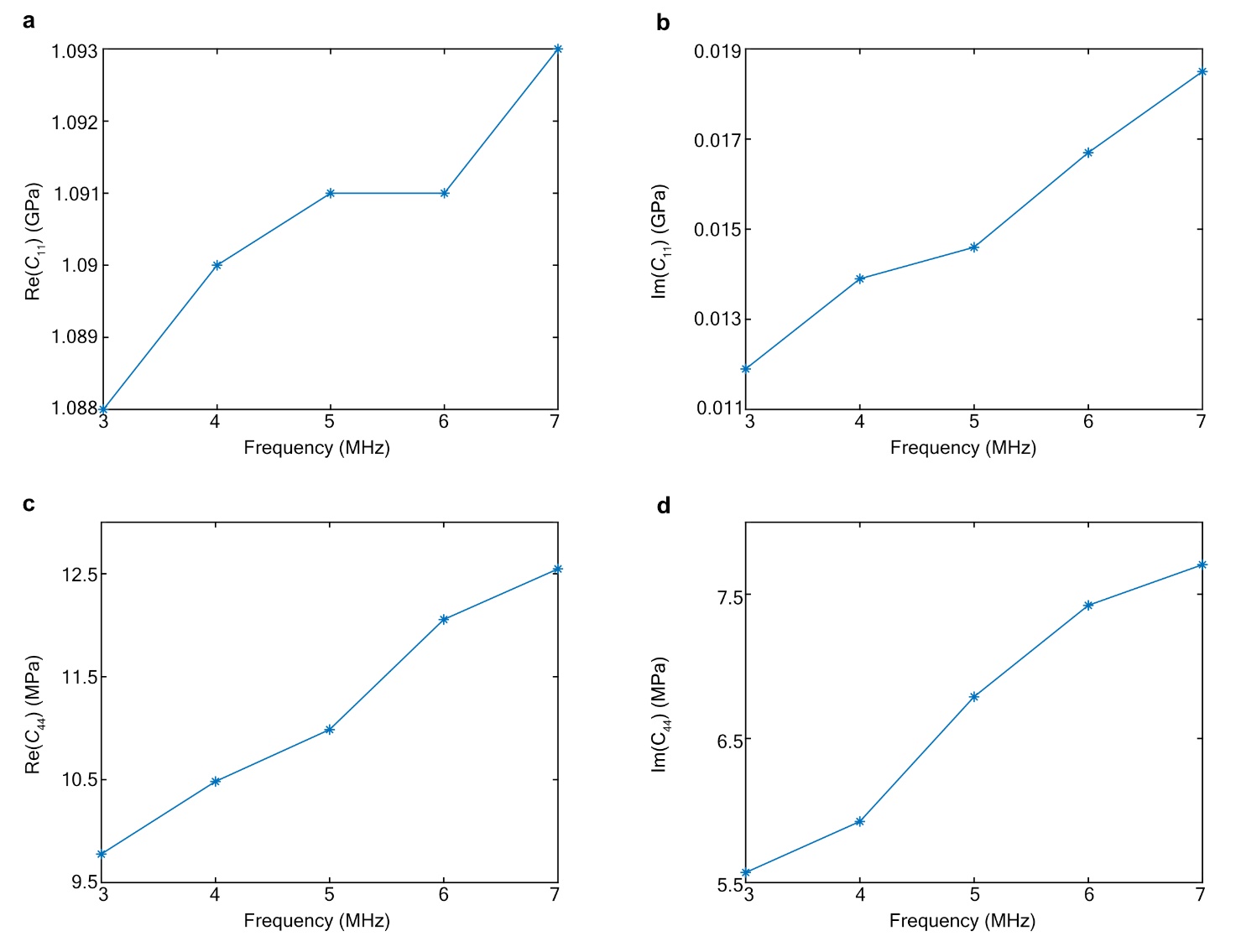


Fig. S1. Frequency dependent elastic constants interpolation. Interpolated values from Xu *et al.*^2^ of the independent elastic constants as function of frequency in PDMS. Real (a-c) and imaginary part (b-d) of $\boldsymbol{C}_{\mathbf{11}}$ and $\boldsymbol{C}_{\mathbf{44}}$.


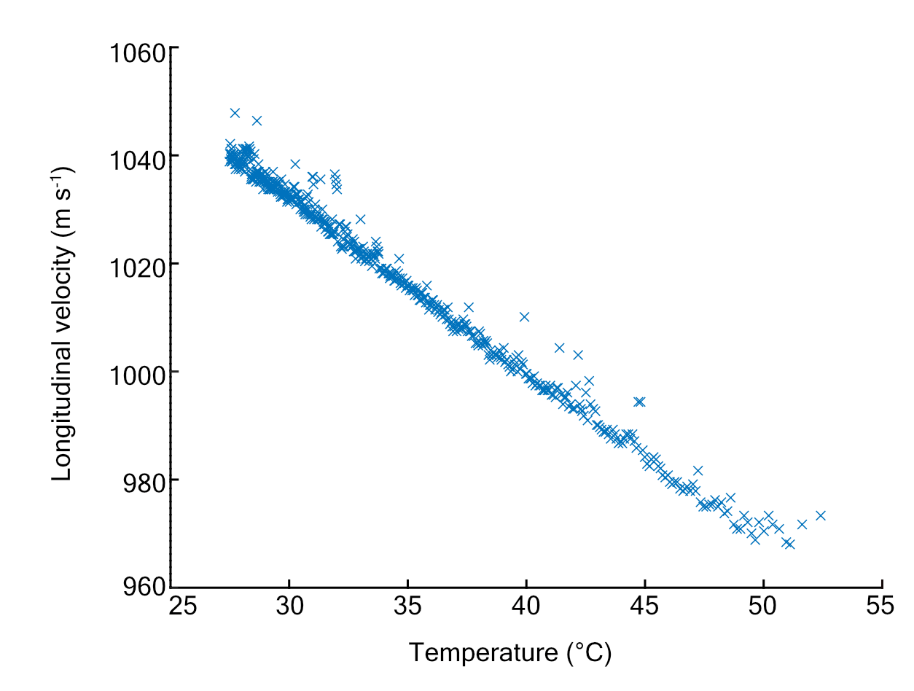


**Fig. S2. Experimental values of longitudinal speed of sound of bulk PDMS, at different temperatures.**


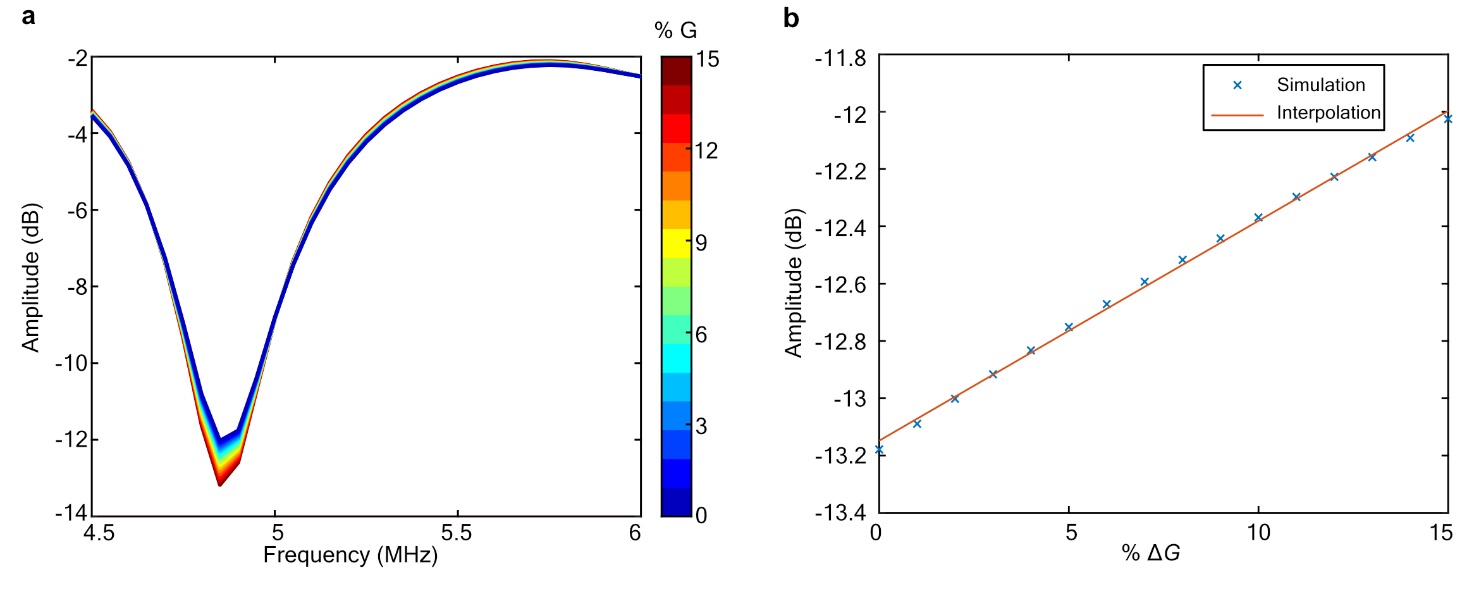


**Fig. S3. FEM simulated results with shear modulus percentile variation.** (**a**) Simulated amplitude of the reflection spectrum in the PDMS-Meta for different values of percentage variation of shear modulus. (**b**) Linear interpolation (red, continuous line) for modulus minimum value (blue x) for each percentage variation of the shear modulus (sensitivity).

**
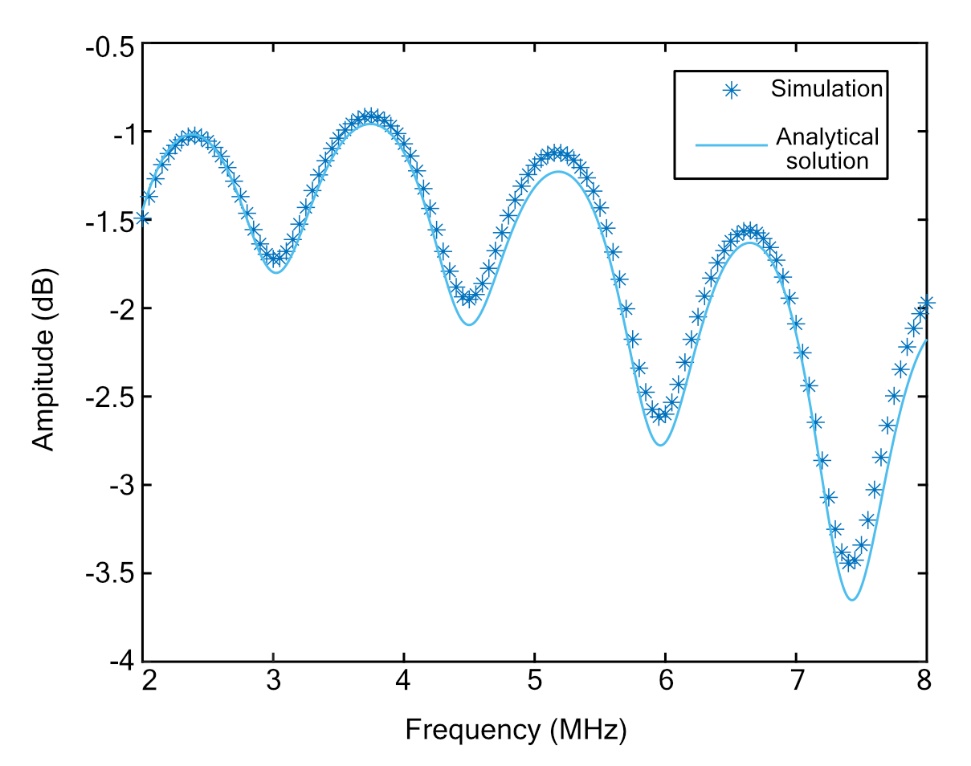
**

.

**Fig. S4. Analytical vs simulated reflection spectrum for Bilayer structure.** Comparison between the analytical solution of the bilayer (continuous line) vs the simulated unit cell in water (asterisks) in Comsol Multiphysics. Thickness PDMS: $350 \mu m$.


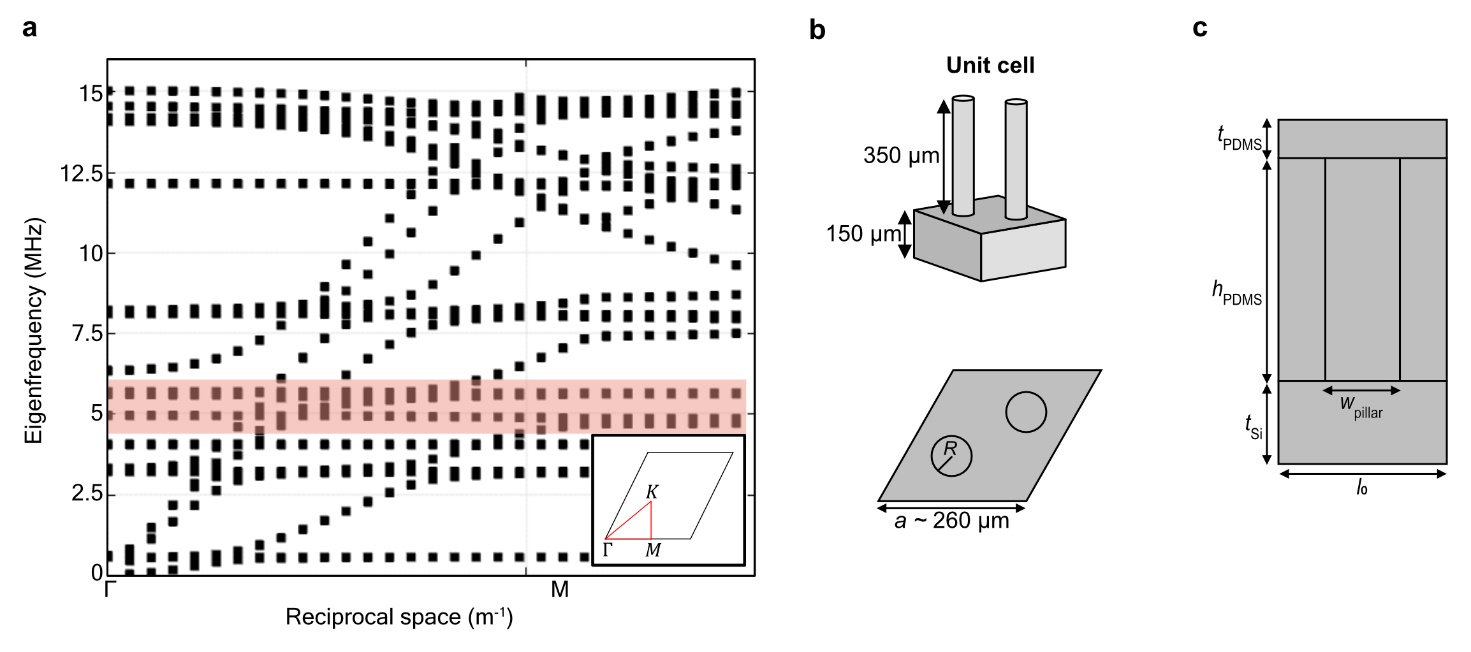


Fig. S5. Computed band structure of 3D Si-Meta and unit cells implemented in the simulations. (a) Computed band structure at FEM methods of the 3D Si-Meta from an elementary unit cell. Highlighted in red: eigenfrequencies corresponding to standing waves in the metamaterial (group velocity: $\boldsymbol{v}_{\mathbf{g}}\boldsymbol{=}\frac{\boldsymbol{\partial\omega}}{\boldsymbol{\delta k}}\boldsymbol{=0}$) . A schematic of the unit cell in the reciprocal space is shown in the inset. (b) The unit cell was dimensioned to satisfy the coupling condition between the incoming wave and the PDMS. The characteristic wavelength ($\boldsymbol{\lambda}_{\mathbf{c}}$) in the material was set as design parameter for the unit cell dimensioning: $\boldsymbol{a=}\sqrt{\boldsymbol{3}}\boldsymbol{\cdot}\frac{\boldsymbol{\lambda}_{\mathbf{c}}}{\boldsymbol{3}}\boldsymbol{=260.}\boldsymbol{96 \mu}$m. (c) 2D unit cell used in the FEM mechanic-acoustic simulations for the PDMS-Meta. The width of the equivalent pillar ($\boldsymbol{w}_{\mathbf{pillar}}$) has been adjusted to match the reflection spectrum of the 3D metamaterial.


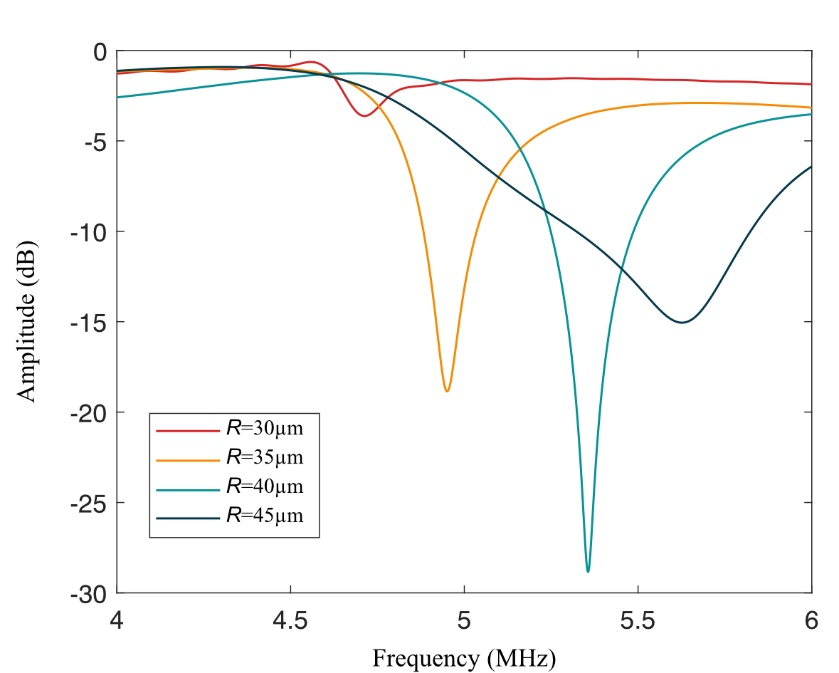


**Fig. S6. Experimental reflection spectra of designs having different radius.** Experimental reflection spectrum of four different design of the Si-Meta sensor with a different pillar radius (*R*). By tuning the radius, it is possible to control the peak position in the frequency domain and match the specifications of the excitation transducer (5 MHz in this work, $R=35\mu m$).


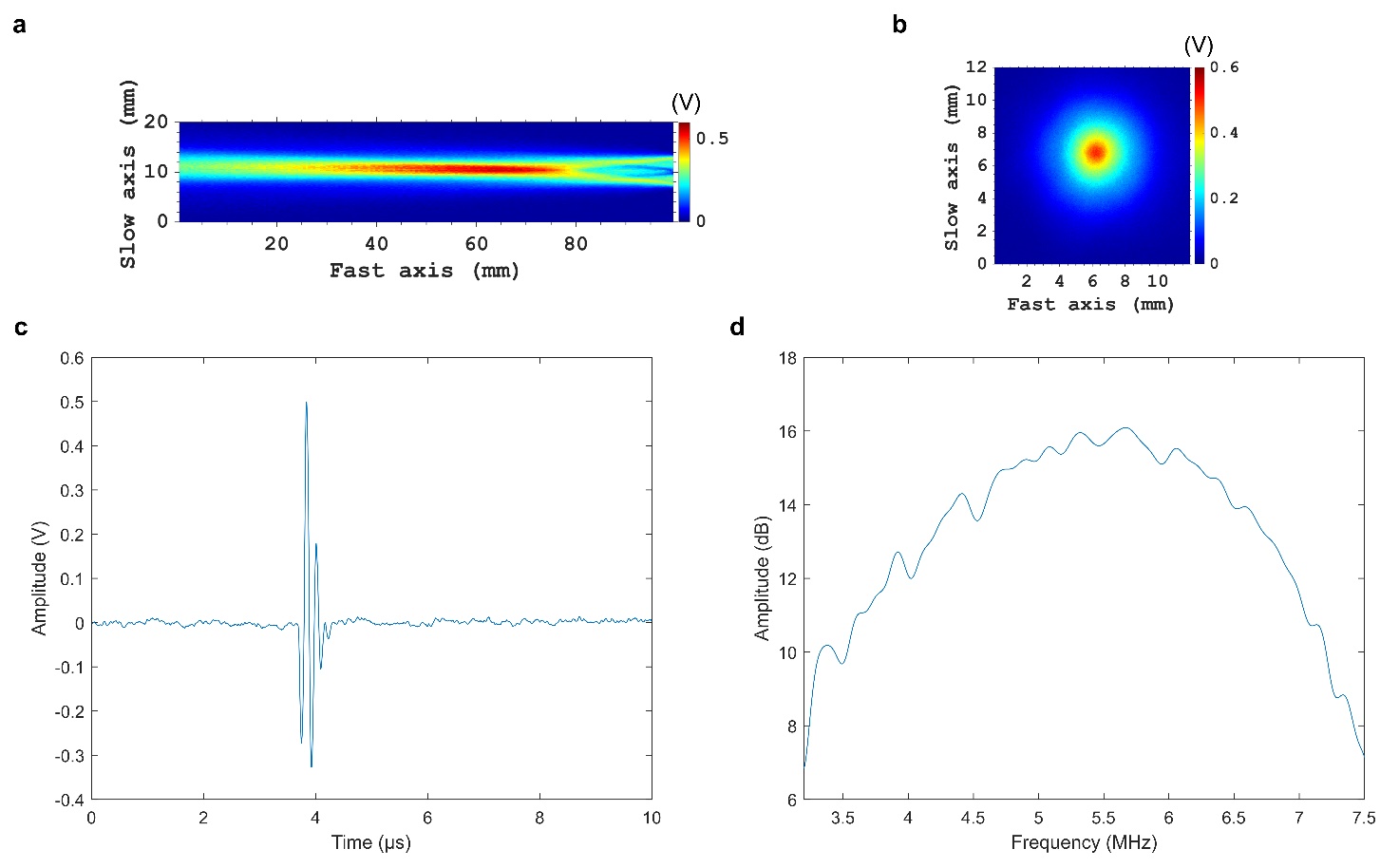


**Fig. S7. Experimental incoming sound field.** Recorded sound field of the excitation ultrasonic pulse (units: V) of ISL-0502-HR probe, with an Onda Hydrophone GL-0200, Pre-Amplifier Onda AG-2010. (**a**) Y-X sound field, where Y=100 mm is close to the transducer and Z-X representation; (**b**) sound field detail at approximate 32mm distance. (**c**) Time trace of the incident time signal (central pixel, duration $\sim1 \mu s$). (**d**) FFT of the incident Gaussian pulse.


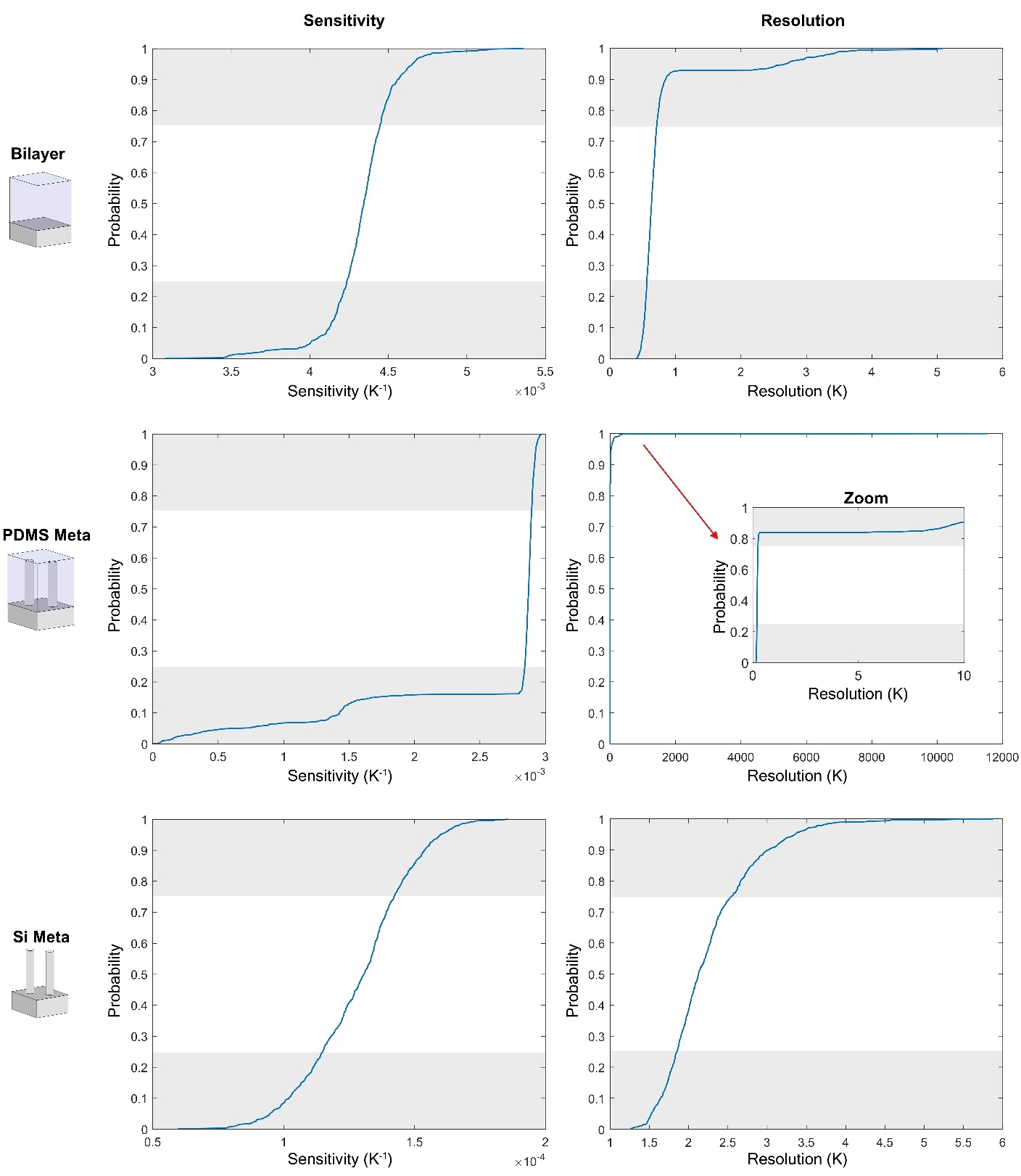


**Fig. S8.**  **Empirical cumulative distribution function (ECDF) of the sensor designs.** ECDF was computed with respect to temperature sensitivity measurements (left) and resolution (right). The grey band represents the atypical pixels excluded from the computation of temperature sensitivity and temperature resolution. A low (high) value for the sensitivity (resolution) was assigned to those pixels, as illustrated in the resolution calculation for the PDMS-Meta: the inset clarifies the typical pixel distribution for this particular sensor. The probability range of typical pixels was defined between $25\%\div75\%$.


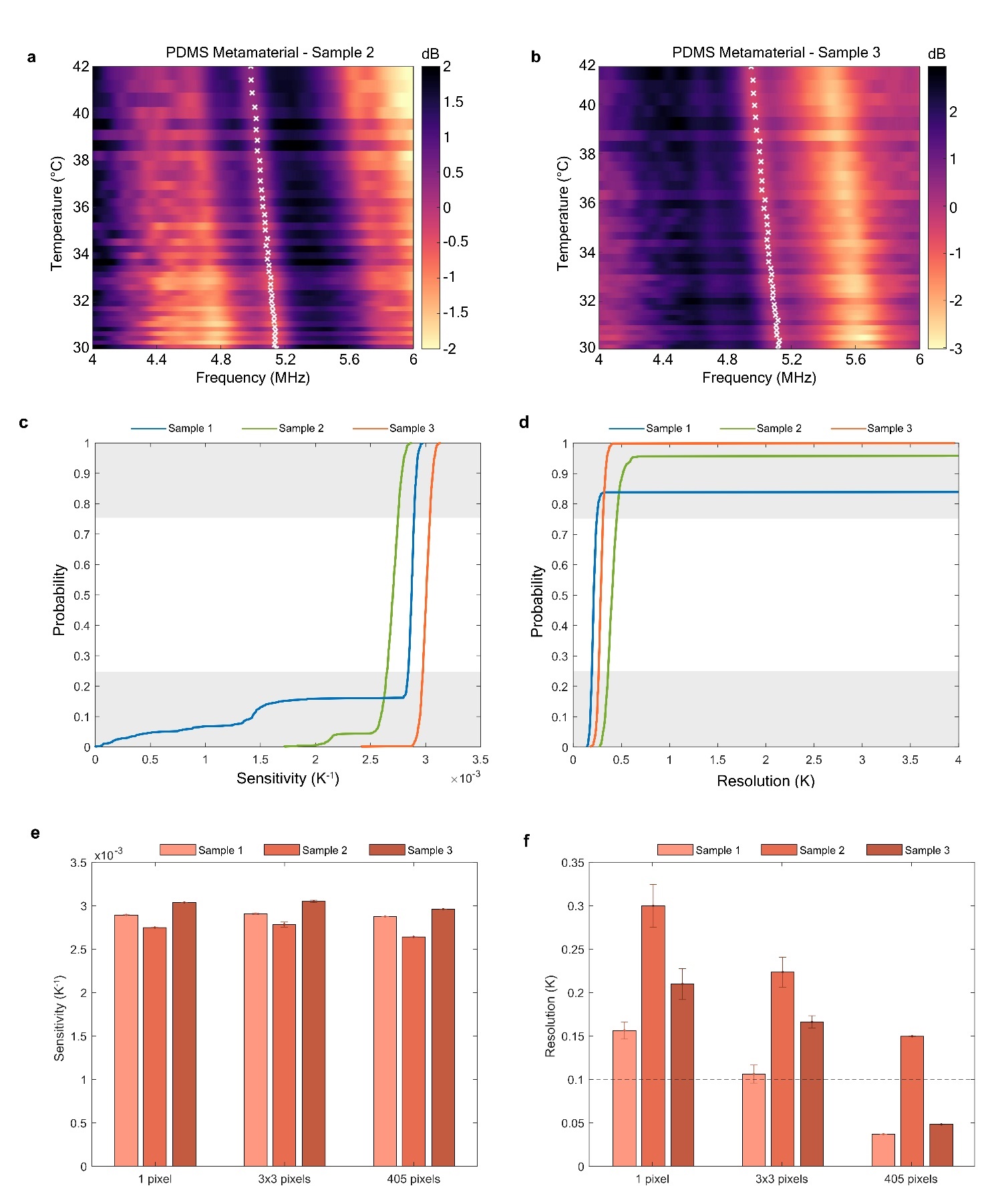


**Fig. S9. Repetition of experiments with two additional PDMS Metamaterial samples.** (**a**) and (**b**) show the temperature dependent FFTs signals for Sample 2 and 3 as 2D maps (see also Fig. 3 in the main text for Sample 1). Computed ECDF for resolution (**c**) and sensitivity (**d**), compared to the sample (Sample 1) in the main text (see also Fig. S8). Comparison of sensitivity (**e**) and resolution (**f**) values of the three samples, with their standard deviation at pixel level, with 3x3 pixels averaging and with average over 405 typical pixels (see also Fig. 4 in the main text for a comparison of PDMS-Meta Sample 1 with a Si-Meta and Bilayer samples).


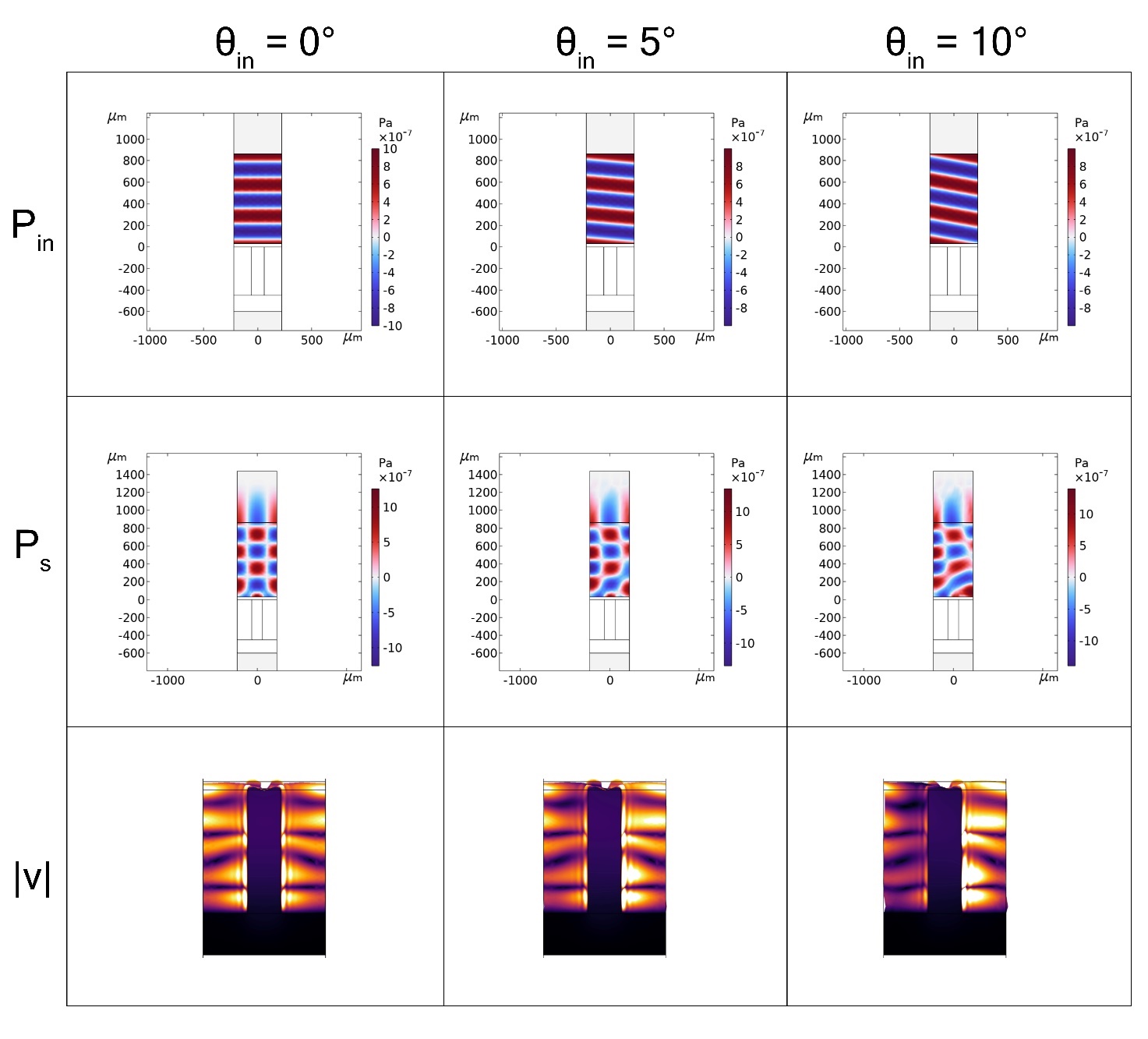


**Fig. S10. Simulated pressure and displacement fields for different directions of incident angle ($\boldsymbol{\theta}_{\mathbf{in}}$) at excitation frequency close to resonance: $\mathbf{f=5.15 MHz}$.** The incident pressure field ($P_{\mathrm{in}}$) is shown at the top; in the middle, the reflected pressure field ($P_{s}$) and at the bottom the absolute value of the vertical displacement (|v|). The vertical displacement shows an asymmetric distribution of the displacement field in the metamaterial, with an increased value of $\theta_{\mathrm{in}}$.


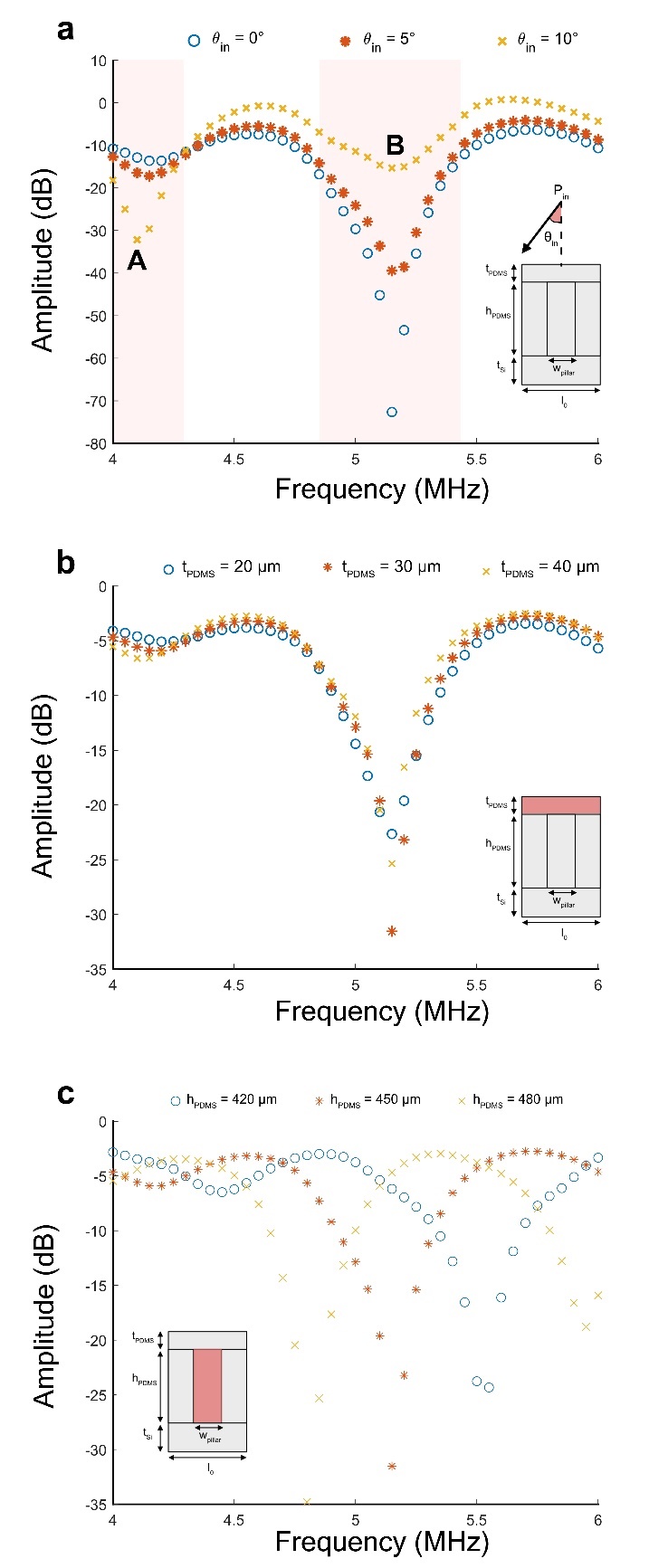


**Fig. S11. Simulated reflection spectrum of PDMS Meta with fabrication process variations.** Reflection spectrum resulting from different values of: (**a**) incident angle $\theta_{\mathrm{in}}$; (**b**) top layer PDMS thickness $t_{\mathrm{PDMS}}$; (**c**) height of pillar $l_{\mathrm{pillar}}\boldsymbol{\equiv}h_{\mathrm{PDMS}}$. While (b) modestly contributes to a modulation of the amplitude of the reflection spectrum, the incident angle plays a major role, inducing a strong reduction of the typical resonance peak B of the metamaterial around 5 MHz. Furthermore, peaks outside the range of interest, e.g. peak A, are amplified as result of interferences due to artifacts introduced by the incident angle. Also the modulation of the height of the pillars affects the position of the resonance peak, as displayed in (c).


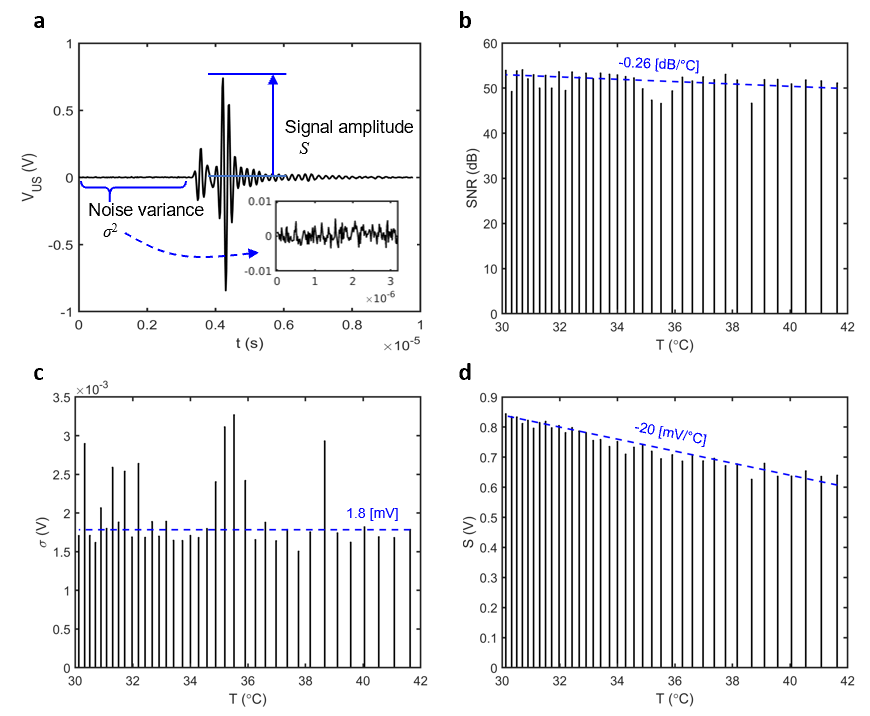


**Fig. S12. Signal-to-Noise Ratio (SNR) temperature-dependence analysis for pixel (3,44) of the PDMS Meta Sample 1. (a)** an echo signal (time-domain) at 37°C, used to define the Signal amplitude S (peak absolute value) and noise variance *σ*^2^. The inset shows a close-up of the region utilized to calculate *σ* which consists of noise (no signal present). (**b**) SNR shows a weak dependence on temperature (slope ~ -0.26dB/°C) and has a median value of 52dB over all temperature values. (**c**) Noise standard deviation does not show a marked temperature dependence (median value 1.8mV). (**d**) Reflected signal amplitude decreases with increasing temperature, with a slope of -20mV/°C.


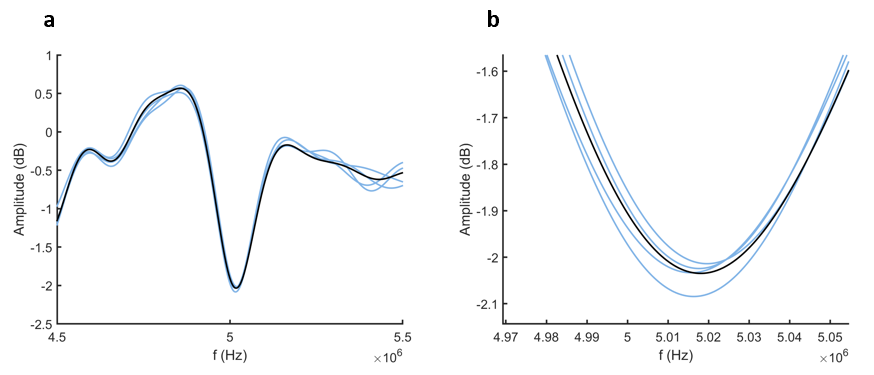


**Fig. S13. Simulated effect of soft-tissue attenuation on resonance peak of PDMS-Meta.
(a,b)** Measured (black line) and four examples of simulated (blue lines) amplitude spectra of the PDMS Meta Sample 1. (**b**) is a close-up of (**a**) in the region of the 5.02 MHz resonance peak of the PDMS Meta. Simulation means injecting noise into signals (additive Gaussian noise in the time-domain) to match the expected SNR degradation from the attenuation of soft-tissue at 5 MHz (~-6dB for 1cm thick tissue)

**Supplementary Table 1. Experimental parameters of P/E equipment.**

| **Sensor** | Bilayer | PDMS-Meta | Si-Meta |
| --- | --- | --- | --- |
| **Attenuation (dB)** | 11 | 3 | 18 |

The measurements were acquired at constant gain (40 dB), energy (1 μJ), and damping (50 $\Omega$). Filter characteristics: low-pass (LP) 1 kHz; high-pass (HP): 50 MHz.

**Supplementary Table 2. Customized materials properties and expressions for the FEM simulation**

**a. Silicon**^5,6^

| Density ($\mathrm{kg}m^{-3}$) | Young’s modulus (GPa) | Poisson’s ratio |
| --- | --- | --- |
| 2320 | 169 | 0.22 |

**b. PDMS**^2^

| Density ($\mathrm{kg}m^{-3}$) | 1030 |
| --- | --- |
| Bulk modulus | $\hat{B}\left(f\right)=\hat{C}_{11}\left(f\right)-\frac{4}{3}\hat{C}_{44}\left(f\right)+\alpha\cdot\left(\hat{C}_{11}\left(f\right)-\frac{4}{3}\hat{C}_{44}\left(f\right)\right)$ |
| Shear modulus | $\hat{G}\left(f\right)=\hat{C}_{44}\left(f\right)+\beta\cdot\hat{C}_{44}\left(f\right)$ |

**Supplementary Table 3. Geometrical dimensions of unit cells (2D).**

| Sensor | Bilayer | Si-Meta | PDMS-Meta |
| --- | --- | --- | --- |
| $t_{\mathrm{PDMS}} (\mu m)$ | - | - | $30$ |
| $h_{\mathrm{PDMS}}$ $(\mu m)$ | $450$ | $450$ | $450$ |
| $t_{\mathrm{Si}}$ $(\mu m)$ | $150$ | $150$ | $150$ |
| $w_{\mathrm{pillar}}$ $(\mu m)$ | - | $120$ | $120$ |
| $l_{0}$ $(\mu m)$ | $445$ | $445$ | $445$ |

**Supplementary Table 4. Summary table comparing resolution of PDMS Meta without simulated attenuation and with simulated attenuation equivalent to soft tissue (-6 dB):**

| Quantity | Attenuation = 0 dB | | Simulated attenuation = -6 dB | |
| --- | --- | --- | --- | --- |
| Averaging | 1 pixel | 405 pixels | 1 pixel | 405 pixels |
| Resolution (K) | 0.16 | $30\cdot{10}^{-3}$ | 0.23 | $43\cdot{10}^{-3}$ |

Movie S1.

Bilayer.mp4: Absolute value of vertical displacement field in the Bilayer sensor simulated in Comsol at the resonance frequency value: 𝑓=4.6 MHz for Δ𝐵=0% .

**Movie S2.**

Si-Meta.mp4: Absolute value of vertical displacement field in the Si-Meta sensor simulated in Comsol at the resonance frequency value: 𝑓=5.1 MHz for Δ𝐵=0%.

**Movie S3.**

PDMS-Meta.mp4: Absolute value of vertical displacement field in the PDMS-Meta sensor simulated in Comsol at the resonance frequency value: 𝑓= 5.2 MHz for Δ𝐵=0%.

**References**

1. Skov, N. R., Sehgal, P., Kirby, B. J. & Bruus, H. Three-dimensional numerical modeling of surface-acoustic-wave devices: Acoustophoresis of micro-and nanoparticles including streaming. *Phys. Rev. Appl.* **12**, 44028 (2019).

2. Xu, G. *et al.* Acoustic characterization of polydimethylsiloxane for microscale acoustofluidics. *Phys. Rev. Appl.* **13**, 54069 (2020).

3. Brekhovskikh, L. & Godin, O. *ACOUSTICS OF LAYERED MEDIA I*. (Springer- Verlag Berlin Heidelberg New York, 1990).

4. Brandner, D. M., Cai, X., Foiret, J., Ferrara, K. W. & Zagar, B. G. Estimation of tissue attenuation from ultrasonic B-mode images—Spectral-log-difference and method-of-moments algorithms compared. *Sensors* **21**, 2548 (2021).

5. Dolbow, J. & Gosz, M. Effect of out-of-plane properties of a polyimide film on the stress fields in microelectronic structures. *Mech. Mater.* **23**, 311–321 (1996).

6. Hopcroft, M. A., Nix, W. D. & Kenny, T. W. What is the Young’s Modulus of Silicon? *J. microelectromechanical Syst.* **19**, 229–238 (2010).
